# Genetic Dissection of Antibiotic Adjuvant Activity

**DOI:** 10.1128/mbio.03084-21

**Published:** 2022-01-18

**Authors:** J. Bailey, L. Gallagher, W. T. Barker, V. B. Hubble, J. Gasper, C. Melander, C. Manoil

**Affiliations:** a Department of Genome Sciences, University of Washington, Seattle, Washington, USA; b Departments of Chemistry and Biochemistry, University of Notre Dame, Notre Dame, Indiana, USA; McMaster University

**Keywords:** *Acinetobacter*, Tn-seq, aminoimidazole, avibactam, *baumannii*, *baylyi*, meropenem, vancomycin

## Abstract

Small molecule adjuvants that enhance the activity of established antibiotics represent promising agents in the battle against antibiotic resistance. Adjuvants generally act by inhibiting antibiotic resistance processes, and specifying the process acted on is a critical step in defining an adjuvant’s mechanism of action. This step is typically carried out biochemically by identifying molecules that bind adjuvants and then inferring their roles in resistance. Here, we present a complementary genetic strategy based on identifying mutations that both sensitize cells to antibiotic and make them “adjuvant blind.” We tested the approach in Acinetobacter baumannii AB5075 using two adjuvants: a well-characterized β-lactamase inhibitor (avibactam) and a compound enhancing outer membrane permeability (aryl 2-aminoimidazole AI-1). The avibactam studies showed that the adjuvant potentiated one β-lactam (ceftazidime) through action on a single β-lactamase (GES-14) and a second (meropenem) by targeting two different enzymes (GES-14 and OXA-23). Mutations impairing disulfide bond formation (DsbAB) also reduced potentiation, possibly by impairing β-lactamase folding. Mutations reducing AI-1 potentiation of canonical Gram-positive antibiotics (vancomycin and clarithromycin) blocked lipooligosaccharide (LOS/LPS) synthesis or its acyl modification. The results indicate that LOS-mediated outer membrane impermeability is targeted by the adjuvant and show the importance of acylation in the resistance. As part of the study, we employed Acinetobacter baylyi as a model to verify the generality of the A. baumannii results and identified the principal resistance genes for ceftazidime, meropenem, vancomycin, and clarithromycin in A. baumannii AB5075. Overall, the work provides a foundation for analyzing adjuvant action using a comprehensive genetic approach.

## INTRODUCTION

Several alternatives to traditional antibiotic discovery have been proposed for confronting the antibiotic resistance crisis, including the development of adjuvants that increase the efficacy of established drugs (1–6). Resistance to an antibiotic is often distinguished as to whether its mechanism is “acquired” and “intrinsic,” with acquired resistance typically corresponding to dedicated detoxifying functions like β-lactamases encoded in the accessory genome, and intrinsic resistance being more general and encoded in the core genome, such as that due to the outer membrane permeability barrier in Gram-negative bacteria. Adjuvants can target either type of resistance. For example, a variety of β-lactamase inhibitors and outer membrane barrier-compromising compounds have been identified (7–11).

Natural product whole-cell screening has been fruitful in the unbiased discovery of new antibiotic adjuvants (3, 4). A key step in such studies is defining how an adjuvant sensitizes bacteria to an established antibiotic, i.e., defining the resistance mechanism it compromises. The principal methods used to reach this goal have been biochemical, based on identifying adjuvant-binding proteins and inferring their roles in resistance (12–14). While this strategy has markedly increased our understanding of adjuvant mechanisms, it has potential limitations, e.g., that knowing the identity of an adjuvant-binding molecule does not guarantee that the actual resistance process it functions in is obvious, that some of the biochemical methods depend on the previous identification of candidate target molecules for purification and further analysis, and that some of the methods assume that binding targets are proteins. In the study presented here, we developed a complementary strategy that bypasses these limitations by identifying mutations that compromise adjuvant-targeted resistance processes themselves.

Our study focused on Acinetobacter baumannii, an ESKAPE pathogen notorious for its expression of multiple, often redundant antibiotic resistance determinants (15–18). We employed A. baumannii AB5075, a highly virulent isolate exhibiting robust antibiotic resistance which has been developed as a genetically manipulable strain representative of current clinical isolates (19). AB5075 encodes an extended spectrum class A β-lactamase (GES-14), two class D oxacillinases (OXA-23 and OXA-69), a class C enzyme (AmpC), and several β-lactamase relatives (20–24). The GES-14 gene is in a resistance island (RI-2) carried on a plasmid, whereas the others are chromosomal (20, 25). AB5075 produces lipooligosaccharide (LOS) in place of lipopolysaccharide (LPS) as the principal outer membrane permeability barrier (26–28). LOS is nonessential under some growth conditions but is needed for resistance to multiple antibiotics (16). Like many A. baumannii strains, AB5075 undergoes a phase variation that affects resistance and other traits, apparently due in part to differences in capsule production in the variant types (29, 30). The strain also undergoes a high-frequency gene duplication that produces unstable aminoglycoside resistance (31). Resources available for AB5075 include an arrayed transposon mutant library, transposon insertion sequencing (Tn-seq) technology, and a list of genes essential for growth (19, 20, 31–36). In addition, an extensive Tn-seq analysis of antibiotic resistance in a different A. baumannii strain (ATCC 17978) provides a general reference for the species (17). We also employed A. baylyi to test the generality of findings for A. baumannii. A. baylyi strain ADP1 is an antibiotic sensitive relative of A. baumannii that has been developed as a model for synthetic biology because of its high DNA transformation competence (37–39).

**FIG 1:**
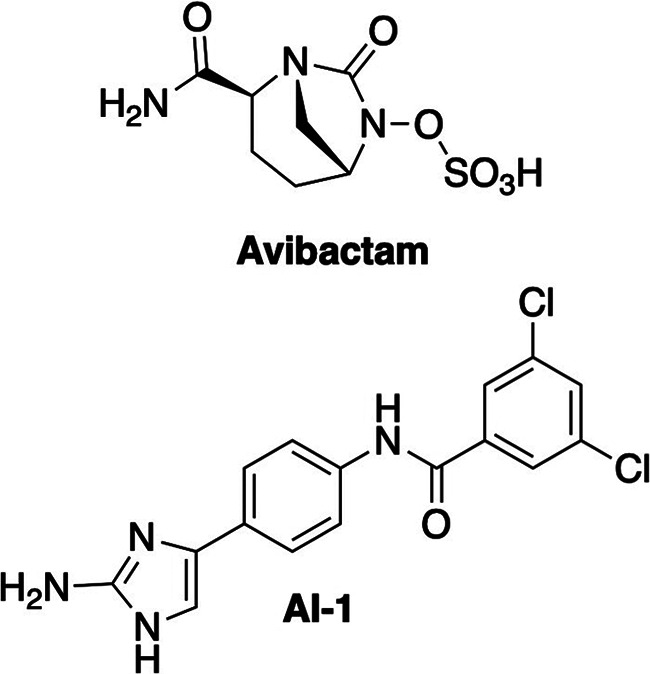
Antibiotic adjuvants employed in this study. Avibactam is a β-lactamase inhibitor of the diazabicyclo-octane class, and AI-1 is an aryl 2-aminoimidazole thought to enhance outer membrane permeability.

Here, we developed a genetic procedure to identify adjuvant target processes and evaluated it using two adjuvants targeting different resistance mechanisms (Fig. 1). The first adjuvant studied was avibactam, a well-characterized broad spectrum β-lactamase inhibitor active against most class A and some class D enzymes (7). The compound potentiates the activity of multiple β-lactam antibiotics against A. baumannii (40, 41). The second adjuvant studied was aryl 2-aminoimidazole AI-1, a compound which compromises the A. baumannii outer membrane permeability barrier. The adjuvant potentiates the activities of antibiotics normally ineffective against Gram-negative species, including the glycopeptide vancomycin and the macrolide clarithromycin, as well as several β-lactams (10, 42). The specific binding target of AI-1 is not known, but the adjuvant is presumed to compromise the outer membrane permeability barrier because of the drugs it potentiates, because it fails to potentiate a colistin-resistant mutant and because it alters LOS structure (10).

## RESULTS AND DISCUSSION

### Rationale.

**FIG 2:**
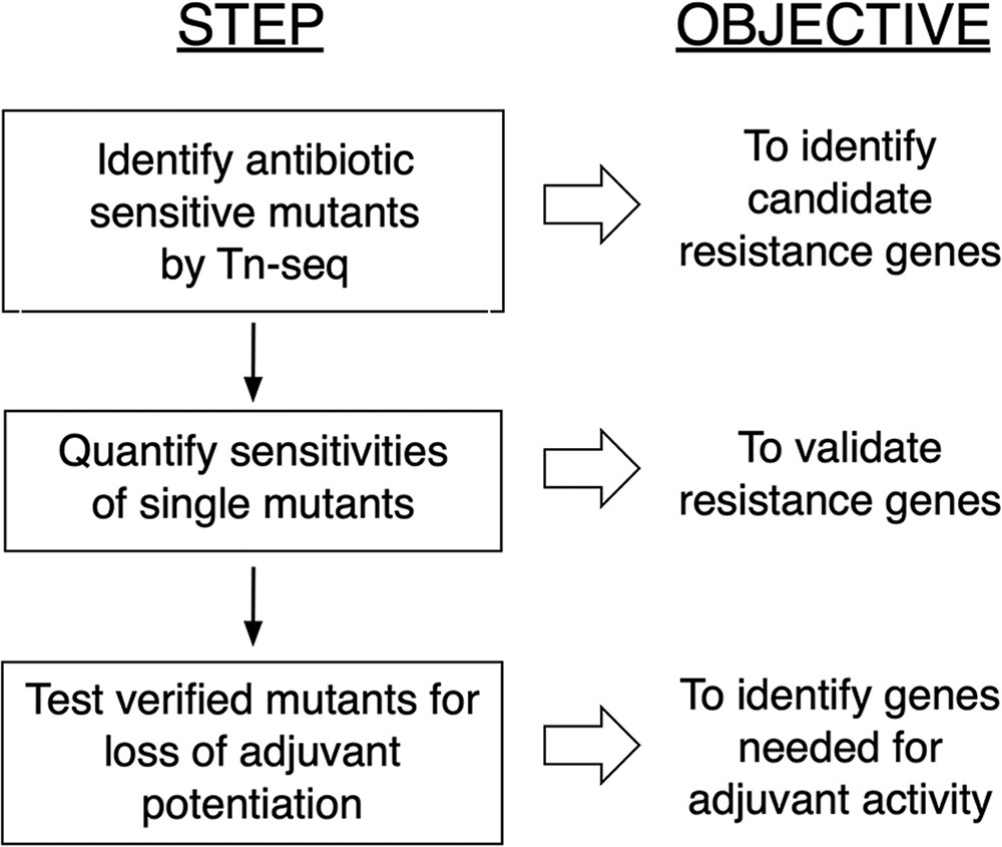
Genetic identification of resistance processes targeted by antibiotic adjuvants. The approach is based on identifying mutations that mimic (phenocopy) treatment with adjuvant in enhancing antibiotic sensitivity. The approach assumes that among all mutations sensitizing bacteria to a potentiated antibiotic, the subset that is not further sensitized by adjuvant inactivates the targeted resistance process. The genes identified by this procedure should in principle include both those encoding molecules binding adjuvant and auxiliary functions needed for the binding target to function.

The primary goal of this study was to develop and test a general genetic approach for identifying the resistance processes targeted by antibiotic adjuvants. The assumption underlying the approach is that mutations inactivating an adjuvant-targeted resistance mechanism will both create a sensitivity phenotype mimicking exposure to the adjuvant and cause loss of adjuvant potentiation. We implemented the approach in three steps (Fig. 2). First, we identified the resistance determinants for an antibiotic potentiated by an adjuvant of interest at genome scale by transposon insertion sequencing (Tn-seq). We next examined candidate resistance loci one-by-one to validate and quantify their mutant sensitivity phenotypes. We then screened validated antibiotic-sensitive mutants for reduced antibiotic potentiation by adjuvant. Mutations sensitizing cells to an antibiotic and eliminating potentiation of its activity by adjuvant should inactivate the resistance process targeted by the adjuvant. In addition to actual adjuvant-binding targets, gene products needed for the binding target to function in resistance should meet these criteria. To evaluate this approach, we analyzed adjuvants acting on genetically simple (β-lactamase) or complex (outer membrane impermeability) resistance processes in A. baumannii AB5075.

### Avibactam studies.

As a proof-of-concept study, we analyzed avibactam. Avibactam enhances the activities of the β-lactams ceftazidime (a third-generation cephalosporin) and meropenem (a carbapenem) (see Fig. S1) against A. baumannii (40, 41), and we examined both. The approach outlined in Fig. 2 predicts that one or more of the four principle β-lactamases encoded by AB5075 will behave as avibactam targets for the antibiotics.

10.1128/mBio.03084-21.1 FIG S1 Structures of the antibiotics employed in this study. Download FIG S1, TIF file, 2.5 MB. Copyright © 2022 Bailey et al. 2022 Bailey et al. https://creativecommons.org/licenses/by/4.0/ This content is distributed under the terms of the Creative Commons Attribution 4.0 International license.

### Avibactam-ceftazidime.

We first identified mutations increasing ceftazidime sensitivity using Tn-seq. Screens were carried out at three ceftazidime concentrations considerably lower (>16-fold) than the AB5075 minimal growth inhibitory concentration (MIC) (>2,048 μg/mL) to identify preferentially mutants with strong sensitivity phenotypes. The top hits identified in the screen corresponded to the genes for the GES-14 β-lactamase (*bla*_GES-14_), a replication function of the plasmid carrying the GES-14 gene (*repA*), and a glucose-inhibited division protein (*gidA*) (see Table S1, top) (43). GES-14 was previously implicated in ceftazidime resistance in a different strain of A. baumannii (23). Insertions in the three other β-lactamase genes (*bla*_OXA-23_, *bla*_OXA-69_, and *ampC*) did not increase sensitivity (see Table S1 bottom and not shown). Mutations inactivating two genes contributing strongly to meropenem resistance (see below), corresponding to an LOS transport function (*lptE*) and a function needed for disulfide bond formation (*dsbA*), exhibited weak ceftazidime sensitivity phenotypes in Tn-seq, with depletion only at the highest antibiotic concentration assayed (see Table S1, bottom).

10.1128/mBio.03084-21.2 TABLE S1 Ceftazidime-sensitive mutants identified by Tn-seq. The genes with the strongest mutant ceftazidime sensitivity phenotypes based on loss after growth in normally subinhibitory ceftazidime are listed at the top. Genes with weaker mutant meropenem sensitivity phenotypes or encoding β-lactamases are included below. The top ceftazidime sensitivity genes correspond to those significantly depleted (*P* < 0.05) in the assays at 32 μg/mL and 64 μg/mL ceftazidime, with a minimum of 100 reads and length of at least 100 residues. A gene that has scored as essential in most other assays and narrowly exceeded the minimum read cutoff here (*nrdB*) was not included. Download Table S1, DOCX file, 0.02 MB. Copyright © 2022 Bailey et al. 2022 Bailey et al. https://creativecommons.org/licenses/by/4.0/ This content is distributed under the terms of the Creative Commons Attribution 4.0 International license.

A concern in interpreting transposon mutant phenotypes is that insertions may have polar effects on transcriptionally downstream genes which affect phenotypes. For most mutants with antibiotic sensitivity phenotypes analyzed in this study, significant polar effects are unlikely either because downstream genes are absent or because the saturation-level genome coverage in the Tn-seq screens ruled out such mutant sensitivities for the genes. A detailed description of potential polar effects on antibiotic sensitivities of the top mutants analyzed in this study is provided in Materials and Methods.

We validated the ceftazidime Tn-seq findings by examining individual transposon mutants from the AB5075 arrayed transposon mutant library (20) and newly constructed deletion mutants (Materials and Methods). GES-14 inactivation strongly sensitized cells to ceftazidime, reducing the MIC >100-fold in the absence of avibactam. However, mutations in *gidA* had no discernible effect (Table 1), and mutations in *repA* were not tested because they were absent from the mutant library. We suspect that *gidA* mutations lead to a weak sensitivity phenotype detectable in Tn-seq experiments, in which mutants are grown in competition, but which is not strong enough to be seen in single mutant assays. Mutations in *lptE* and *dsbA* partially enhanced sensitivity. Mutations inactivating β-lactamase OXA-23 alone did not significantly increase ceftazidime sensitivity, and a GES-14 OXA-23 double mutant was no more sensitive than the GES-14 single mutant. The results imply that the GES-14 β-lactamase is the principal ceftazidime resistance function and that outer membrane LOS and disulfide bond formation are needed for full resistance.

**TABLE 1:** Avibactam potentiation of ceftazidime action is eliminated by β-lactamase GES-14 mutations^*a*^

Gen+e	Mutation	Product	Ceftazidime MIC (μg/mL)		ΔMIC
			–avibactam	+avibactam	
–	None	–	>2,048	14 ± 2	>146
*trpB*	Insertions		>2,048	12	>170
*bla*_GES-14_	Deletion	β-Lactamase GES-14	11 ± 2	7.5 ± 1	1.5
*lptE*	Insertions	LOS transport	136 ± 57	0.9 ± 0.2	151
*dsbA*	Insertions	Thiol:disulfide interchange	717 ± 108	12 ± 5	59
*gidA*	Insertions	Division	>2,048		
*bla*_OXA-23_	Deletion	β-Lactamase OXA-23	>2,048	12	>170
*bla*_GES-14_, *bla*_OXA-23_	Deletions	β-Lactamases GES-14 and OXA-23	12	8	1.2

a Mutants were assayed for ceftazidime sensitivity in the presence and absence of avibactam (64 μg/mL). The values reflect 4 to 13 independent efficiency of plating assays of multiple alleles, and nonzero sample standard deviations are shown. The *trpB* mutants serve as wild-type transposon-containing strains. A deletion mutant lacking resistance island 2, which includes *bla*_GES-14_, gave MIC values comparable to the Δ*bla*_GES-14_ single mutant (not shown). LOS, lipooligosaccharide; MIC, minimal growth inhibitory concentration

We then examined avibactam potentiation of ceftazidime activity for the mutants with increased sensitivities. Avibactam greatly sensitized wild-type strains, OXA-23 and *lptE* mutants to ceftazidime (ΔMIC >146-fold), indicating that its resistance target was still active in these strains. In contrast, avibactam had almost no effect on the ceftazidime sensitivities of the GES-14 single mutant and the GES-14 OXA-23 double mutant (ΔMICs <2-fold) (Table 1). The results indicate that GES-14 is the main target of avibactam in potentiating ceftazidime activity. Avibactam also showed reduced ceftazidime potentiation of *dsbA* mutants (Table 1). The result suggests that DsbA could contribute to ceftazidime resistance by acting on the primary avibactam target GES-14, e.g., by promoting formation of the enzyme’s disulfide bond and stabilizing its folded structure (44).

### Avibactam-meropenem.

We next examined avibactam potentiation of meropenem activity. We carried out Tn-seq screens at a wide range of normally subinhibitory meropenem concentrations (seven screens at 2- to 10-fold below the AB5075 MIC) in order to identify both strong and weak resistance functions (see Table S2). A total of 37 genes showed significant mutant depletion, and the genes could be grouped into classes based on strength of phenotype. By far the strongest sensitization was seen for insertions in LOS transport function *lptE*, which unlike most other LOS synthetic genes, was not essential under the Tn-seq growth conditions employed (20). We suspect that *lptE* mutations only partially impair LOS transport, as has been observed in other bacteria (45, 46). Other top meropenem sensitive mutants (classes 1 and 2 in Table S2) inactivated the genes for β-lactamase OXA-23, LOS modification enzymes (*lpxL* and *lpsB*), capsule synthesis genes (*wzy*, *wzb*, and *wzc*), a capsule biosynthetic enzyme also involved in LOS synthesis (*gna*) (47), and genes needed for disulfide bond formation (*dsbAB*), zinc transport (*znuABC* and *zurA*), regulation (*rpoE* and *rseP*), or peptidoglycan metabolism (*pbpG* and *ampG*). For the three β-lactamases other than OXA-23, only mutations inactivating GES-14 increased meropenem sensitivity, although the phenotype was relatively weak (class 4).

10.1128/mBio.03084-21.3 TABLE S2 Meropenem sensitive mutants identified by Tn-seq. The genes and corresponding sequence read recoveries for growth on LB agar containing meropenem relative to agar without meropenem are shown for seven independent Tn-seq assays at the meropenem concentrations indicated. Mutants were grouped into four classes based on meropenem sensitivity with the following probability cutoffs: class 1, depleted in 2/2 4 μg/mL assays; class 2, depleted in 1/2 4 μg/mL and ≥2/3 6 μg/mL assays; class 3, depleted in ≥2/3 6 μg/mL assays only; class 4, depleted in 1-2/3 6 μg/mL assays only but associated with peptidoglycan synthesis or protein secretion. The probability cutoffs used were *P* < 0.01 (4 μg/mL meropenem [trial 1], and 6 μg/mL meropenem [trials 1 and 2]), and *P* < 0.05 (4 μg/mL meropenem [trial 2] and 6 μg/mL meropenem [trial 3]). Several genes exhibited meropenem sensitive mutant phenotypes but were not included because they were slow growing in the absence of antibiotic and missed the essentiality cutoff (*ompA*, *ompR*, *rpoH*, and *rlpA*). Download Table S2, DOCX file, 0.02 MB. Copyright © 2022 Bailey et al. 2022 Bailey et al. https://creativecommons.org/licenses/by/4.0/ This content is distributed under the terms of the Creative Commons Attribution 4.0 International license.

Tn-seq findings were verified by assaying individual transposon mutants (Table 2). As expected, *lptE* mutations led to the greatest increase in meropenem sensitivity seen, reducing the MIC >12-fold minus avibactam. β-lactamase OXA-23 mutations reduced the MIC 4-fold. Other genes with verified mutant sensitivities included *dsbAB*, *gna*, *pbpG*, and *znuA*, as well as additional genes needed for LOS synthesis (*lpsB* and *lpxL*), regulation (*rpoE* and *ompR*), peptidoglycan metabolism (*ampG*), or of unknown function (ABUW_0466) (48, 49). These mutations presumably define the most significant AB5075 meropenem resistance functions.

**TABLE 2:** Meropenem sensitivity and avibactam potentiation of transposon mutants^*a*^

Gene	Function	Tn-seq class	Insertion site (bp)^*b*^	Meropenem MIC (μg/mL)		ΔMIC
				–avibactam	+avibactam	
Wild type	–	–	–	12 ± 0.8	0.25	48
*trpB*	Tryptophan synthesis	–	381 (1,230)	12	0.25	48
			670 (1,230)	11 ± 1	0.25	44
*bla*_OXA-23_	β-Lactamase OXA-23	1	121 (822)	3	0.09	33
			230 (822)	3	0.09	33
*lptE*	LOS synthesis	1	18 (510)	0.67 ± 0.3	0.009	74
			229 (510)	0.31 ± 0.12	0.008	39
			384 (510)	0.25	0.008	32
			404 (510)	0.38	0.008	48
*pbpG*	Peptidoglycan metabolism	1	277 (1,008)	3.5 ± 0.6	0.11 ± 0.02	32
			140 (1,008)	4	0.09	44
*znuA*	Zinc transport	1	391 (840)	6	0.12	50
			211 (840)	6	0.12	50
*gna*	Capsule synthesis	1	319 (1,275)	3.5 ± 0.6	0.03	117
			157 (1,275)	4	0.06	67
*dsbA*	Disulfide formation	1	336 (618)	3.5 ± 0.7	–	–
			184 (618)	3.8 ± 0.5	0.19	20
			64 (618)	4	0.16 ± 0.04	25
*dsbB*	Disulfide formation	2	56 (516)	6	0.25	24
			106 (516)	5 ± 1.4	0.25	20
*ampG*	Peptidoglycan recycling	2	460 (2,190)	4	0.09	44
			779 (2,190)	4	0.09	44
ABUW_0466	Unknown	2	320 (663)	6	0.25	24
			470 (663)	6	0.25	24
*rpoE*	Sigma factor	2	236 (615)	6	0.09	67
*lpxL*	LOS synthesis	2	314 (936)	3	0.05	60
*lpsB*	LOS synthesis	2	333 (1,101)	4	0.05	80
			742 (1,101)	2.5 ± 2.1	0.04 ± 0.01	63
*bla*_GES-14_	β-lactamase GES-14	4	378 (864)	11 ± 1.4	0.25	44
*ompA*	Outer membrane protein	–	103 (1,062)	4	–^*c*^	–
			943 (1,062)	3	–^*c*^	–
*ompR*	Two-component regulation	–	502 (765)	2.75 ± 0.5	0.05	55
			411 (765)	2.5 ± 0.6	0.05	50
*envZ*	Two-component regulation	4	888 (1,458)	6	–^*c*^	–

a The average values of two to seven efficiency-of-plating assays for each mutant are presented. Nonzero sample standard deviations are shown. A complete list of mutants identified by Tn-seq is shown in Table S2, including their class assignments based on depletion after growth in the presence of meropenem. Additional genes with verified but smaller individual mutant meropenem sensitivities (minus avibactam) (ΔMIC 6 to 10) were *qhbB* (capsule synthesis); *bfmR*, *rseP*, and *dksA* (regulation); *mrdA*, *dacC*, *elsL*, and *rlpA* (peptidoglycan synthesis); ABUW_0460 (unknown function); and *znuC* (zinc transport). The *ompA* and *ompR* genes showed low mutant read recovery in Tn-seq and were not assigned a class.

b Transposon insertion position in gene (gene length).

c Sensitive to avibactam alone.

The role of the ZnuABC zinc transport system in meropenem resistance is uncertain. However, recent work identified a zinc limitation-induced d,d-carboxypeptidase (ZrlA) which provides a potential link to peptidoglycan metabolism (50).

Meropenem activity against wild type and *trpB* transposon mutant control strains was highly potentiated by avibactam (ΔMICs ≥ 44-fold) (Table 2). LptE mutants were equally potentiated by the adjuvant (ΔMIC ≥ 32-fold), indicating that the avibactam target was still functional in the LOS transport mutant (Table 2). Somewhat surprisingly, OXA-23 deletion mutants also showed considerable potentiation by avibactam (ΔMIC = 33-fold). None of 14 other meropenem-sensitive mutations fully eliminated the potentiation either, although three (in *dsbA*, *dsbB*, and ABUW_0466) reduced it partially (ΔMICs = 20- to 25-fold). The results suggest that multiple targets could contribute to avibactam potentiation of meropenem activity, and thus no single mutation would fully eliminate it. We tested this possibility in two ways: by examining AB5075 double mutants and by transferring genes into A. baylyi.

Since β-lactamase GES-14 contributed detectably to meropenem resistance in a wild-type AB5075 genetic background (Table 2; see also Table S2), we examined whether it was responsible for the residual resistance and avibactam potentiation of OXA-23 mutants. We generated single and double deletion mutants of GES-14 and OXA-23 and examined their meropenem sensitivities ± avibactam. Strains carrying a deletion of the 13.5-kbp resistance island (RI-2) that includes the GES-14 gene were also included. We found that double mutants lacking both GES-14 and OXA-23 β-lactamase genes were much more sensitive to meropenem than the corresponding single mutants in the absence of avibactam and were not further sensitized by avibactam (Table 3). The results indicate that OXA-23 and GES-14 contribute redundantly to meropenem resistance, with OXA-23 dominating when both enzymes are present, and that both enzymes are inhibited by avibactam.

**TABLE 3:** Meropenem sensitivity and avibactam potentiation in deletion mutants and transplant derivatives^*a*^

Mutation(s)	No. of isolates tested	Meropenem MIC (μg/mL)		ΔMIC
		–avibactam	+avibactam	
*A. baumannii*				
None	1	12	0.25	48
Δ*bla*_OXA-23_	2	4	0.094	43
Δ*bla*_GES-14_	3	8	0.25	32
ΔRI–2	1	8	0.25	32
Δ*bla*_OXA-23_ Δ*bla*_GES-14_	3	0.094	0.094	1
Δ*bla*_OXA-23_ ΔRI-2	1	0.094	0.094	1
				
*A. baylyi*				
None	1	0.064	0.031	2
+ *kan*	1	0.064	0.047	1.4
+ *bla*_OXA-23_	2	8	0.19	42
+ *bla*_GES-14_	2	0.75	0.047	16
+ *bla*_OXA-23_ + *bla*_GES-14_	2	10	0.19	52

a MIC values are based on duplicate efficiency of plating assays in LB of independently derived strains. The sample standard deviations were <5% in all cases. The slight (≤2-fold) avibactam potentiation consistently seen for wild-type A. baylyi was eliminated by a mutation inactivating penicillin binding protein 2 (which is not essential in A. baylyi), suggesting that the protein contributes somewhat to the potentiation (not shown). An A. baylyi mutant deleted of *lptE* reduced the meropenem MIC 12-fold in a *bla*-minus background and 48-fold in a transplant strain expressing OXA-23 and GES-14, indicating that the *lptE*-minus sensitivity phenotype is independent of the two β-lactamases. RI-2, resistant island 2 (contains *bla*_GES-14_).

**FIG 3:**
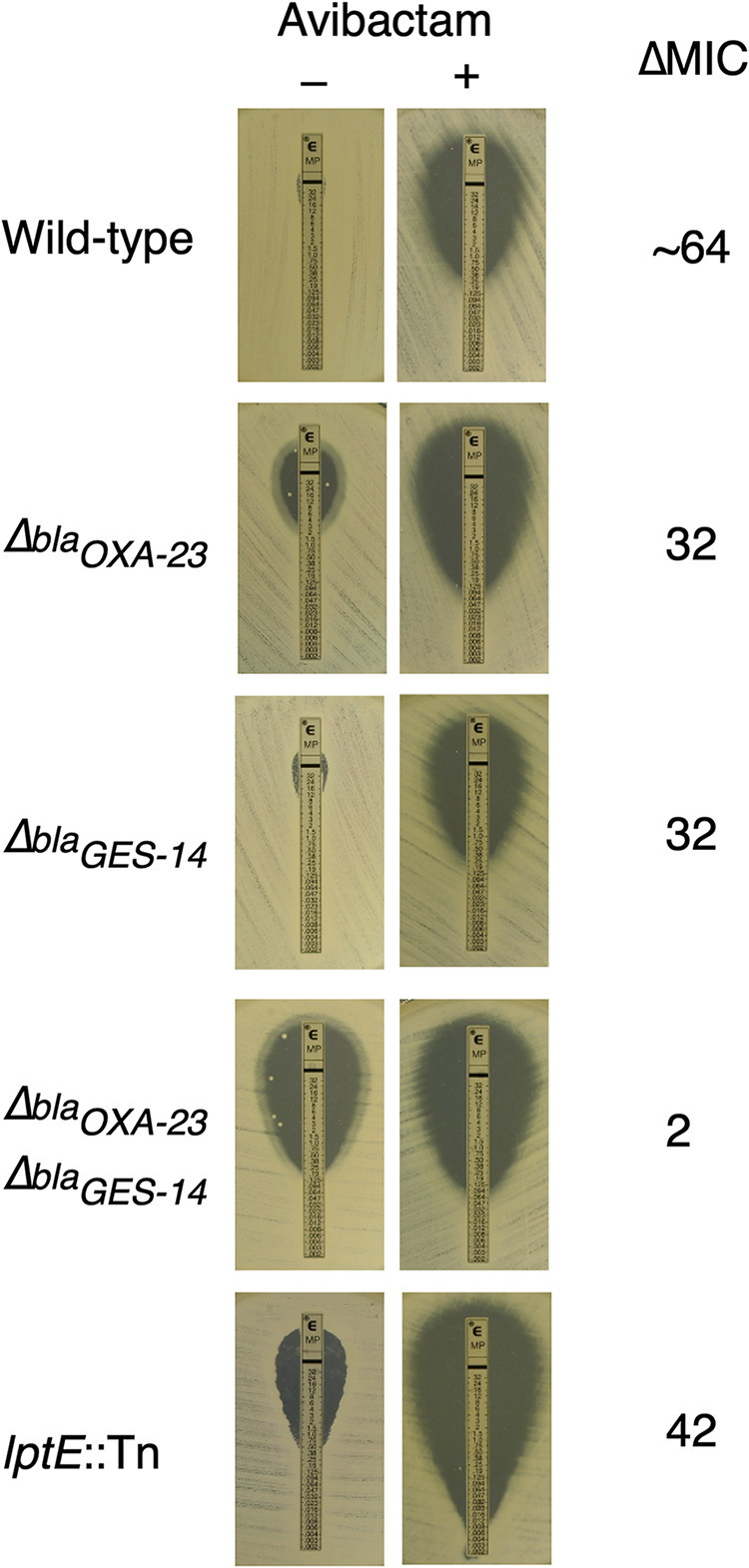
Adjuvant potentiation plate test. The image shows meropenem sensitivity ± avibactam using bacteria grown overnight on LB agar in the presence of Etest strips. The approximate MICs based on these assays (–avibactam, + avibactam in μg/mL) were as follows: wild-type (24, 0.38), *Δbla*_OXA-23_ (4, 0.125), *Δbla*_GES-14_ (12, 0.38), *Δbla*_OXA-23_ *Δbla*_GES-14_ (0.25,0.125), *lptE*::Tn (0.5, 0.012). LB agar was supplemented where indicated with 64 μg/mL avibactam.

The contributions of both OXA-23 and GES-14 to avibactam potentiation of meropenem activity can be readily observed by the growth of wild-type and mutants on agar ± avibactam with meropenem Etest strips (Fig. 3). The zone of clearing is dramatically greater with avibactam in the agar medium for the wild-type, the two single β-lactamase mutants and the LptE mutant (ΔMIC = 32- to 64-fold). However, the OXA-23-GES-14 double mutant exhibits only modest potentiation by the adjuvant (ΔMIC = 2-fold).

To further examine the apparent redundancy in β-lactamases acting on meropenem, we examined “transplant” strains in which the GES-14 and OXA-23 genes were inserted into the genome of A. baylyi. A. baylyi is a genetically manipulable relative of A. baumannii which does not exhibit significant β-lactam resistance (51). The properties of the transplant strains reflected those of the corresponding A. baumannii mutants (Table 3). Compared to the parent and wild-type control strains, the OXA-23 single transplant increased the meropenem MIC 10-fold more than the GES-14 transplant, and the double transplant was slightly more resistant than the OXA-23 single transplant. Both individual transplants and the double transplant were potentiated by avibactam, whereas the parent and control strain showed little potentiation. Taken together, these findings confirm that the OXA-23 and GES-14 β-lactamases almost entirely account for AB5075 meropenem resistance and that avibactam sensitizes cells by inhibiting both. One possible explanation for the unusual redundancy phenotype is that OXA-23 interaction with one or more outer membrane porins transporting meropenem provides a kinetic advantage over GES-14 in the inactivation of the antibiotic (33).

### Summary of avibactam findings.

The results of the avibactam analysis help validate the approach outlined in Fig. 2 for identifying a relatively simple class of adjuvant target (β-lactamases) and show that the approach can succeed in the face of target redundancy. The non β-lactamase genes for which mutants show partially reduced potentiation by avibactam, namely, *dsbAB* and ABUW_0466, may act indirectly to facilitate folding or export of the target β-lactamases.

### Aryl 2-aminoimidazole AI-1 studies.

We next examined adjuvant AI-1 (Fig. 1), which sensitizes A. baumannii to antibiotics normally ineffective against Gram-negative species, including the macrolide clarithromycin and the glycopeptide vancomycin (see Fig. S1) (10). The adjuvant is thought to compromise the outer membrane permeability barrier, allowing the potentiated antibiotics to reach their cellular targets.

We first screened for AB5075 clarithromycin or vancomycin hypersensitive mutants by Tn-seq. We identified 15 genes showing strong mutant depletion with vancomycin and nine with clarithromycin, with six genes in common (see Table S3). Three of the genes in common function in LOS synthesis or modification (*lptE*, *lpsB*, and *gna*), fitting with the key role of LOS in outer membrane impermeability to the drugs. Most other genes involved in LOS production were poorly represented in the transposon mutant pools analyzed due to their essentiality under the Tn-seq screening conditions employed (20) and could not be evaluated. We thus assume that the three LOS mutations identified lead to only partial loss of the outer membrane permeability barrier and can be tolerated. Mutants not known to be associated with LOS synthesis with strong sensitivity phenotypes for the antibiotics affect synthesis of peptidoglycan (*rlpA*), capsule (*gtr51*), or metabolism (*glnA*) (see Table S3). Overall, the results are consistent with the tenet that the two antibiotics are normally ineffective against Gram-negative bacteria due to poor outer membrane permeation.

10.1128/mBio.03084-21.4 TABLE S3 Vancomycin and clarithromycin sensitive mutants identified using Tn-seq. Genes with greatest depletion of transposon insertion reads following growth in the presence of normally subinhibitory vancomycin or clarithromycin are shown. Genes identified in both screens are in boldface. The genes listed correspond to those showing strongest mutant depletion (lowest log sequence read ratios) after growth in the levels of antibiotic shown relative to no antibiotic (≥2/3 *P* < 0.01). vanc, vancomycin; clar, clarithromycin. Download Table S3, DOCX file, 0.02 MB. Copyright © 2022 Bailey et al. 2022 Bailey et al. https://creativecommons.org/licenses/by/4.0/ This content is distributed under the terms of the Creative Commons Attribution 4.0 International license.

The next steps of the analysis were validation of clarithromycin- and vancomycin-sensitive phenotypes with single mutants, followed by screens for loss of adjuvant potentiation (Fig. 2). However, in the process of carrying out these studies, we found that the growth of many mutants of interest was inhibited by adjuvant AI-1 alone. To identify adjuvant-sensitive mutants at a comprehensive scale, we carried out Tn-seq screens in the presence of AI-1 alone (see Table S4). The screens identified mutations in 27 genes that significantly increased AI-1 sensitivity, including genes required for capsule synthesis (10 genes), phospholipid retrograde transport (four genes), and LOS core oligosaccharide synthesis (four genes). The results suggest that reducing the capsule or LOS oligosaccharide permeability barriers or increasing the phospholipid content of the outer membrane sensitizes cells to adjuvant. A strong LOS-minus mutation (*lpxC*::*ISAba1*) remains sensitive to adjuvant (see below), indicating that the toxicity is not due to an effect on LOS.

10.1128/mBio.03084-21.5 TABLE S4 Mutants sensitive to adjuvant AI-1 in Tn-seq. Genes showing depletion of transposon insertion sequence reads following growth in the presence of normally subinhibitory adjuvant AI-1 (LB containing 20 μM AI-1) in two independent trials are listed. The genes listed correspond to those showing greatest mutant depletion after growth with adjuvant relative to without adjuvant (*P* < 0.01 in both trials) for the log sequence read ratios in Tn-seq. Genes ABUW_3816 to ABUW_3832 are K locus genes involved in extracellular capsule production. Download Table S4, DOCX file, 0.02 MB. Copyright © 2022 Bailey et al. 2022 Bailey et al. https://creativecommons.org/licenses/by/4.0/ This content is distributed under the terms of the Creative Commons Attribution 4.0 International license.

To bypass the adjuvant toxicity complication, we focused further analysis on three genes whose mutants were sensitive to vancomycin and/or clarithromycin but were insensitive to adjuvant alone. The genes encode functions required for LOS transport (*lptE*), LOS acyl modification (*lpxL*) (52, 53), and a chaperone (*dnaJ*). We retrieved individual mutants for the three genes from the arrayed AB5075 transposon mutant library and assayed their antibiotic sensitivities. Mutations in all three genes increased sensitivity to vancomycin compared to the parent AB5075 or transposon-insertion control strains (*trpB*), and the *lptE* mutations also increased clarithromycin sensitivity (Table 4). We next examined the additional effect of AI-1 and found that the mutations affecting LOS reduced but did not eliminate potentiation (measured as ΔMIC) of vancomycin (*lptE* and *lpxL*) and clarithromycin (*lptE*), whereas the *dnaJ* mutation had only a small effect on vancomycin potentiation. The results fit with a mechanism in which AI-1 acts on LOS to compromise the outer membrane permeability barrier.

**TABLE 4:** AI-1 potentiation of vancomycin and clarithromycin activity in mutants^*a*^

Gene	Insertion site (bp)	Vancomycin MIC (μM AI-1)				Clarithromycin MIC (μM AI-1)			
		0	15	20	ΔMIC^*b*^	0	15	20	ΔMIC^*a*^
AB5075	–	256	64	32	8	32	4	2	16
*trpB*	381 (1,230)	256	64	32	8	32	2	2	16
	670 (1,230)	384 ± 181	96 ± 45	24 ± 11	16	32	4	2	16
*lptE*	18 (510)	8	2	1.5 ± 0.7	5.3	6 ± 3	1	0.5	12
	229 (510)	8	1	1	8	4	1	0.75 ± 0.4	5.3
*lpxL*	314 (936)	128	48 ± 22	32	4	32	2	2	16
	414 (936)	64	64	32	2	32	2	2	16
*dnaJ*	449 (1,113)	64	16	12 ± 6	5.3	32	3 ± 1	2	16
	628 (1,113)	96 ± 45	16	12 ± 6	8	32	4	2	16
*lpxC*	395 (903)	0.125	0.125	0.125	1	0.0625	0.0625	0.0625	1
	473 (903)	0.25	0.125	0.125	2	0.0625	0.0625	0.062	1

a Values represent means of 3 or 4 assays by broth microdilution in LB containing different levels of AI-1. Non-zero sample standard deviations at 48 hours are shown. The MIC of AI-1 alone was 50 μM under these conditions.

b ΔMIC at 20 μM adjuvant.

Since neither of the LOS mutations eliminated potentiation completely, it was possible that they did not fully compromise the outer membrane permeability barrier, or that functions other than LOS also contribute to potentiation by AI-1. To evaluate this possibility, we isolated 12 mutants expected to be strongly LOS-minus by selecting strong colistin resistance under conditions in which LOS is not essential (54) (see Materials and Methods). Nearly all (11/12) of the mutants isolated carried IS element insertions in *lpxC*, the gene for first committed step of LOS synthesis (52) (see Materials and Methods). Two of these mutants examined were exquisitely sensitive to vancomycin and clarithromycin and showed virtually complete loss of AI-1 potentiation (Table 4). The results imply that LOS function is likely to be the sole significant resistance determinant acted on by AI-1.

We examined AI-1 toxicity toward the adjuvant-blind *lpxC* mutants and found little change from the wild type (e.g., MIC = 75 ± 27 for AB5075 and 70 ± 27 for LpxC^–^ strain MAB203). The finding shows that AI-1 antibiotic potentiation and toxicity are genetically separable and indicates that AI-1 toxicity does not act through LOS.

To examine whether the effects seen for A. baumannii AB5075 potentiation extended to another Acinetobacter species, we examined deletion mutants of A. baylyi. In addition to *lptE* and *lpxL*, we created mutations in *lpxM* (encoding a second LOS acyl transferase) (52, 53) and *lpxA* (encoding the first step of LOS synthesis). The *lpxA* mutant was constructed in a suppressor mutant genetic background (Δ*mlaBCDEF* Δ*pldA*) because of its near essentiality in a wild-type genetic background (55). The wild-type control strain (MAY154) exhibited strong AI-1 potentiation of clarithromycin and vancomycin activity, whereas all the LOS mutations reduced or eliminated potentiation for one or both antibiotics (Table 5). The results with A. baylyi thus reflect those for A. baumannii and suggest that LOS is likely to be a relatively general requirement for AI-1 potentiation in Acinetobacter species. They also further underscore the importance of LOS acyl modification for vancomycin potentiation.

**TABLE 5:** AI-1 potentiation of antibiotic activity in A. baylyi mutants^*a*^

Strain	Genotype	Vancomycin MIC (μM AI-1)				Clarithromycin MIC (μM AI-1)			
		0	15	20	ΔMIC	0	15	20	ΔMIC
MAY116	Wild type	256		16	16	8		0.5	16
MAY156	Δ*lptE*	2		0.5	4	0.25		0.125	2
MAY157	Δ*lpxL*	128		24 ± 9	5.3	8		0.5	16
MAY158	Δ*lpxM*	28 ± 5		16	1.8	4		0.5	8
MAY154	Δ*mlaBCDEF* Δ*pldA*	128	53 ± 18		2.4	2	0.5		4
MAY155	Δ*mlaBCDEF* Δ*pldA* Δ*lpxA*	4	4		1	0.0625	0.0625		1

a Values represent means of two to three broth microdilution assays in LB at 45 to 48 h with nonzero sample standard deviations shown. The MIC of AI-1 alone was ≥50 μM for all strains (not shown). MAY116 and MAY154 carry *nptII* in place of an IS element (see Materials and Methods).

A simple explanation for AI-1 potentiation of vancomycin is that it reduces LOS acylation. The model can account for the reduced hydroxyacyl content of LOS isolated from AI-1-treated cells (10), since LpxL adds a hydroxyacyl group to LOS (53). However, since *lpxL* and *lpxM* mutations increase vancomycin sensitivity much more than clarithromycin sensitivity, whereas AI-1 potentiates both antibiotics robustly, reduced acylation seems unlikely to be the sole mechanism by which AI-1 acts.

### Morphology of AI-1-treated cells.

**FIG 4:**
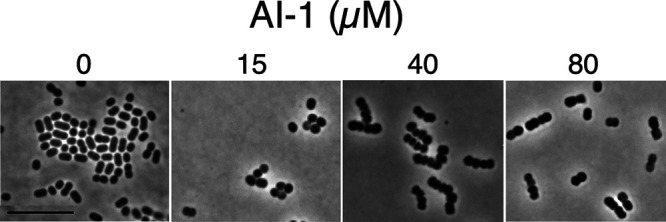
Morphology of AI-1 treated A. baumannii. Bacteria were grown on LB agar containing different levels of AI-1 for 20 h at 37°C and imaged using phase-contrast microscopy. Ratios of 1:2:4 cell chains for the different AI-I concentrations were as follows: 0 μM (0.20:1.0:<0.02), 15 μM (0.13:1.0:0.08), 40 μM (<0.02:1.0:5.0), and 80 μM (0.03:1.0:0.56) (>200 cells counted for each concentration). The MIC for AI-1 is 50 to 75 μM in this medium, which may account for the reduced production of four cell chains at 80 μM.

In previous work with A. baylyi *and* A. baumannii, we and others found that lethal mutations affecting outer membrane biogenesis, including LOS and protein localization, led to a distinctive terminal morphology in which bacteria accumulated as chains of rounded cells (55, 56). The phenotype suggests defects in lateral peptidoglycan synthesis and cell separation. Since AI-1 appears to act on the outer membrane, it should produce a similar morphology. Indeed, growing cells in the presence of AI-1 led to rounding at low concentrations (15 μM) and additional chaining at higher levels (40 and 80 μM) (Fig. 4). The results provide independent support for the conclusion that the adjuvant compromises the outer membrane.

### Summary of AI-1 studies.

The results provide strong support for the hypothesis that AI-1 acts by compromising the LOS-mediated outer membrane permeability barrier, and show the critical role of acyl modification for the barrier function. The study was complicated by the facts that LOS was essential under the conditions used for Tn-seq analysis and that adjuvant alone was toxic to many antibiotic sensitive mutants. Nevertheless, the analysis progressed because partial loss-of-function mutations affecting LOS which were not lethal and did not sensitize cells to adjuvant toxicity could be studied.

### Conclusions.

These studies serve to validate a new, genome-scale approach for defining the resistance mechanisms undermined by antibiotic adjuvants based on identifying loss-of-function mutations that eliminate adjuvant potentiation. The approach was successful in dissecting the simple situation in which the major resistance determinant for an antibiotic was also the function targeted by an adjuvant (avibactam-ceftazidime), but also accommodated more complicated cases in which there were two redundant adjuvant targets (avibactam-meropenem), the target was essential (AI-1), or the adjuvant was toxic (AI-1). In principle, the procedure should identify virtually all the nonessential functions underlying a resistance process targeted by an adjuvant. This genetic approach thus complements biochemical methods focused on defining adjuvant-binding targets based on *in vivo* or *in vitro* affinity (12–14).

The challenges of defining targets for new adjuvants discovered in target-blind screens are not unlike those encountered for new antibiotics, namely, the shortage of general methods available and uncertainty associated with those that are (57). The genetic strategy presented here adds an approach that is general and complementary to established biochemical methods and should thus contribute to the development of this promising class of drugs.

## MATERIALS AND METHODS

### Growth medium, strains, and plasmid.

The growth medium was LB (10 g of tryptone, 5 g of yeast extract, 8 g of NaCl/L with 15 g/L Bacto agar [Difco] for LB agar) at 37°C (A. baumannii) or 30°C (A. baylyi) unless otherwise noted. A. baumannii AB5075-UW (MAB101), transposon T26 insertion pools and single mutants of this strain have been described (20), as has plasmid pSL15a (34). All Acinetobacter strains used for this study are listed in Table 6.

**TABLE 6:** Strains

Strain	Genotype	Locus/loci mutated	Source or reference
*Acinetobacter baumannii*			
AB5075-UW	Wild type		1
MAB103	ΔRI-2	ABUW_4045-4064	2
MAB198	Δ*bla*_OXA-23_	ABUW_0563	This study
MAB199	Δ*bla*_GES-14_	ABUW_4052	This study
MAB200	Δ*bla*_OXA-23_ Δ*bla*_GES-14_	ABUW_0563, ABUW_4052	This study
MAB201	Δ*bla*_OXA-23_ ΔRI-2	ABUW_0563, ABUW_4045-4064	This study
MAB202	Δ*bfmR*	ABUW_3181	This study
MAB203	*lpxC*::ISAba1 at bp 395 of 903	ABUW_0152	This study
MAB204	*lpxC*::ISAba1 at bp 473 of 903	ABUW_0152	This study
			
*Acinetobacter baylyi*			
ADP1	Wild type		3
MAY116	ΔIS1236_1::*nptII*	ACIAD0320-0321	
MAY151	ΔIS1236_1::*bla*_GES-14_ (ABUW_4052)	ACIAD0320-0321	This study
MAY152	ΔIS1236_4::*bla*_OXA-23_ (ABUW_0563)	ACIAD1249-1250	This study
MAY153	ΔIS1236_1::*bla*_GES-14_ ΔIS1236_4::*bla*_OXA-23_	ACIAD0320-0321, ACIAD1249-1250	This study
MAY154	Δ*mlaB-F* Δ*pldA* ΔIS1236_1::*nptII*	ACIAD3241-3245	This study
MAY155	Δ*mlaB-F* Δ*pldA* Δ*lpxA*::*nptII*	ACIAD3241-3245, ACIAD1354	3; this study
MAY156	Δ*lptE*::*aphA6*	ACIAD3107	This study
MAY157	Δ*lpxL*::*aphA6*	ACIAD0484	This study
MAY158	Δ*lpxM*::*aphA6*	ACIAD2638	This study

### Mutant construction and isolation.

A. baumannii nonpolar, unmarked deletion strains were constructed by targeted mutagenesis using a suicide plasmid integration-excision method (34). A fragment consisting of ∼1 kb of flanking sequence on each side of a gene to be deleted was subcloned using added restriction sites into suicide vector, pSL15a. Deletions were designed to be in-frame and remove all but ∼30 bp total from the ends of the genes deleted. The suicide vector was conjugated from either MFDpir or S17-1 into AB5075 or MAB103, with selection for tetracycline (15 μg/mL) and, for S17-1 donor matings, chloramphenicol (10 μg/mL) (58). Tetracycline-resistant cointegrants were purified, streaked onto LB media lacking sodium chloride and containing 6% sucrose, and then incubated at room temperature overnight. In the case of the *bla*_GES-14_ deletion construction, tobramycin at 10 μg/mL was also added to sucrose media to select for p1AB5075 and eliminate sucrose-sensitive colonies that had lost the plasmid. Sucrose-resistant colonies were screened for loss of tetracycline resistance and deletion constructs were confirmed by PCR.

A. baylyi marked and unmarked deletion mutant strains were made through natural transformation of linear fragments as described previously (59), except that flanking sequences used were ∼1 kb in length, the kanamycin resistance marker inserted was the *aphA6* gene from AB5075 (60) and that unmarked deletions did not contain added sequences. All deletion constructs were confirmed by PCR. Insertion strain MAY151 was made by replacement of IS1236_1 sequence (genome bp 321570 to 322805) with the promoter and coding sequences of *bla*_GES-14_ (ABUW_4052) via natural transformation (59). Likewise, MAY152 was made by deletion of IS1236_4 (genome bp 1248193 to 1249429) and insertion of promoter and coding sequences of *bla*_OXA-23_ (ABUW_0563). A double mutant (MAY153) was made via sequential transformations. Primers used for mutant constructions are listed in Table S5.

10.1128/mBio.03084-21.6 TABLE S5 Individual transposon insertion mutants from A. baumannii AB5075 transposon mutant library (20). All alleles are chromosomal except *bla*_GES-14_::T26, which is situated on plasmid p1. Download Table S5, DOCX file, 0.02 MB. Copyright © 2022 Bailey et al. 2022 Bailey et al. https://creativecommons.org/licenses/by/4.0/ This content is distributed under the terms of the Creative Commons Attribution 4.0 International license.

A. baumannii LpxC mutants were isolated by selection for colistin resistance (27, 54). Overnight cultures of AB5075 were plated on LB agar supplemented with colistin (10 μg/mL) and incubated at 37°C overnight. Twelve colistin-resistant colonies found to be vancomycin sensitive were purified, and the *lpxA* and *lpxC* genes sequenced. Eleven of the mutants carried insertions of ISAba1 or ISAba13 in *lpxC* (at four unique locations), and two (MAB203 and MAB204) were studied further (Table 6).

### Antibiotic sensitivity assays.

Three methods were employed for measuring antibiotic minimum growth inhibitory concentrations. For efficiency of plating assays, overnight cultures were diluted and grown to an optical density at 600 nm (OD_600_) of 0.1 to 0.2, and aliquots of serially diluted cultures were spotted (10 μL) on antibiotic plates. After 18 to 24 h of growth, the concentration at which the efficiency of plating fell below 5% was called as the MIC. For strip assays, Etest strips (bioMérieux) were placed on lawns of cells from strains grown as described above prior to plating, and MICs based on growth inhibition around the strip at 18 to 24 h evaluated according to manufacturer’s instructions. In a third method, broth microdilution (10), strains were grown as described above and diluted to 5 × 10^5^ CFU/ml and then distributed in a 2-fold dilution series of antibiotic and grown in a 96-well format for 48 h under stationary conditions in humidified bags. Absorbance measurements were taken with a Tecan SpectroFluor Plus plate reader. For adjuvant potentiation assays, adjuvant was present with cells at appropriate concentration. For AB5075 and its mutants, opaque colony-derived cultures were assayed (32).

### Transposon mutant polar effects.

To evaluate the potential contribution of polar effects to transposon mutant antibiotic sensitivities, we first identified genes with mutant phenotypes with downstream genes defined as co-oriented genes with start codons within 99 bp of the upstream gene termination codon. Of the 23 genes with strong mutant phenotypes shown in Tables 1, 2, and 4, there were five such mutants. Mutations in three of the genes (*gna*, *rpoE*, and *ompR*) may be polar on genes functioning in the same physiological processes (capsule formation, envelope stress response, and two-component regulation, respectively), which could potentially enhance the primary mutant sensitivities. One of the remaining two genes is *ampG*, which may be polar on *gloA* (the gene encoding glyoxylase); however, *gloA* mutants show no increase in meropenem sensitivity in Tn-seq analysis (not shown), indicating that a polar effect on its expression is unlikely to contribute to the *ampG* mutant phenotype. The fifth gene is *lptE*, in which insertions may be polar on expression of *holA*, an essential gene encoding a DNA polymerase holoenzyme subunit. To test whether reduced expression of *holA* was likely to contribute to the *lptE* phenotypes, we constructed an in-frame *lptE* deletion mutant unlikely to show transcriptional polarity in A. baylyi. The deletion mutant sensitivity phenotypes were virtually identical to those of *lptE* insertion mutants (not shown), indicating that polar effects reducing *holA* expression are unlikely to contribute significantly to the *lptE* mutant phenotypes.

### Tn-seq sample preparation and sequence analysis.

For the ceftazidime and meropenem Tn-seq analysis, a pool of ∼450,000 T26 transposon mutagenized strains (20) was thawed, diluted in LB, and grown for an hour with aeration at 37°C prior to diluting and plating on LB agar with subinhibitory levels of antibiotic (ceftazidime at 0, 32, 64, and 128 μg/mL (MIC >2048 μg/mL), and meropenem at 0, 0.5, 1, 2, 4, 6, 9, 12 μg/mL (MIC 12 to 16 μg/mL). After 12 h of growth, colonies were harvested and flash frozen in LB and 10% glycerol prior to Tn-seq processing.

For vancomycin, clarithromycin, and A1-1 Tn-seq analysis, the mutant pool was thawed, diluted into cation-adjusted Mueller-Hinton broth, and subsequently grown in the presence of drugs in 96-well format in deep well blocks (Genetix) at 37°C with aeration. Cells were grown with various subinhibitory levels of drug: vancomycin at 0, 12.2, 18.3, and 30.5 μg/mL (MIC = 256 μg/mL); clarithromycin at 0, 0.418, 0.627, and 1.045 μg/mL (MIC = 32 μg/mL); and A1-1 at 20 μM. After approximately seven population doublings, the cells were pelleted and frozen prior to Tn-seq processing. Genomic DNA was isolated from the transposon mutant pools by DNeasy blood and tissue kit (Qiagen, catalog no. 69506), and ∼6 μg per pool was processed by the terminal deoxynucleotidyl transferase (TdT) method as before (34). The samples were sequenced using a MiSeq (Illumina) with an ∼5% PhiX spike-in and mixed sequencing primers 17 and 18.

Sequence reads were mapped to the AB5075 genome after removing the bases corresponding to the transposon end (reads without transposon sequence were discarded), and read counts were normalized to 10 million total reads per sample (60, 61). Subsequently, reads per gene (between the 5th and 90th percentiles of the open reading frame) were tallied.

### Microscopy.

Microscopy was performed as described previously (59). AB5075 strains were diluted from an overnight, grown at 37°C in LB to an OD_600_ 0.15 and spotted onto a thin pad of LB agar containing AI-1 on microscope slides using Gene Frames (Thermo Scientific). Coverslips were added, and slides were incubated at 37°C. After 20 h of incubation, high-resolution phase-contrast imaging was performed using a 100× oil objective of a Nikon Eclipse 90i microscope. To evaluate ratios of cells in different length chains, images of cells analogous to those shown in Fig. 3 were scored manually focusing on well-isolated individual cells and groups of cells.

10.1128/mBio.03084-21.7 TABLE S6 Oligonucleotide primers. Download Table S6, DOCX file, 0.02 MB. Copyright © 2022 Bailey et al. 2022 Bailey et al. https://creativecommons.org/licenses/by/4.0/ This content is distributed under the terms of the Creative Commons Attribution 4.0 International license.

## References

[1] Farha MA, Brown ED. 2015. Unconventional screening approaches for antibiotic discovery. Ann N Y Acad Sci 1354:54–66. doi:10.1111/nyas.12803. 26100135

[2] Hauser AR, Mecsas J, Moir DT. 2016. Beyond antibiotics: new therapeutic approaches for bacterial infections. Clin Infect Dis 63:89–95. doi:10.1093/cid/ciw200. 27025826 PMC4901866

[3] Wright GD. 2016. Antibiotic adjuvants: rescuing antibiotics from resistance. Trends Microbiol 24:862–871. doi:10.1016/j.tim.2016.06.009. 27430191

[4] Melander RJ, Melander C. 2017. The challenge of overcoming antibiotic resistance: an adjuvant approach? ACS Infect Dis 3:559–563. doi:10.1021/acsinfecdis.7b00071. 28548487 PMC5798239

[5] Theuretzbacher U, Piddock LJV. 2019. Non-traditional antibacterial therapeutic options and challenges. Cell Host Microbe 26:61–72. doi:10.1016/j.chom.2019.06.004. 31295426

[6] Farha MA, Brown ED. 2013. Discovery of antibiotic adjuvants. Nat Biotechnol 31:120–122. doi:10.1038/nbt.2500. 23392510

[7] Bush K, Bradford PA. 2019. Interplay between beta-lactamases and new beta-lactamase inhibitors. Nat Rev Microbiol 17:295–306. doi:10.1038/s41579-019-0159-8. 30837684

[8] Stokes JM, MacNair CR, Ilyas B, French S, Cote JP, Bouwman C, Farha MA, Sieron AO, Whitfield C, Coombes BK, Brown ED. 2017. Pentamidine sensitizes Gram-negative pathogens to antibiotics and overcomes acquired colistin resistance. Nat Microbiol 2:17028. doi:10.1038/nmicrobiol.2017.28. 28263303 PMC5360458

[9] MacNair CR, Brown ED. 2020. Outer membrane disruption overcomes intrinsic, acquired, and spontaneous antibiotic resistance. mBio 11:e01615-20. doi:10.1128/mBio.01615-20. 32963002 PMC7512548

[10] Martin SE, Melander RJ, Brackett CM, Scott AJ, Chandler CE, Nguyen CM, Minrovic BM, Harrill SE, Ernst RK, Manoil C, Melander C. 2019. Small molecule potentiation of Gram-positive selective antibiotics against *Acinetobacter baumannii*. ACS Infect Dis 5:1223–1230. doi:10.1021/acsinfecdis.9b00067. 31002491 PMC6682313

[11] Klobucar K, Cote JP, French S, Borrillo L, Guo ABY, Serrano-Wu MH, Lee KK, Hubbard B, Johnson JW, Gaulin JL, Magolan J, Hung DT, Brown ED. 2021. Chemical screen for vancomycin antagonism uncovers probes of the Gram-negative outer membrane. ACS Chem Biol 16:929–942. doi:10.1021/acschembio.1c00179. 33974796

[12] Milton ME, Minrovic BM, Harris DL, Kang B, Jung D, Lewis CP, Thompson RJ, Melander RJ, Zeng D, Melander C, Cavanagh J. 2018. Re-sensitizing multidrug resistant bacteria to antibiotics by targeting bacterial response regulators: characterization and comparison of interactions between 2-aminoimidazoles and the response regulators BfmR from *Acinetobacter baumannii* and QseB from *Francisella* spp. Front Mol Biosci 5:15. doi:10.3389/fmolb.2018.00015. 29487854 PMC5816815

[13] Thompson RJ, Bobay BG, Stowe SD, Olson AL, Peng L, Su Z, Actis LA, Melander C, Cavanagh J. 2012. Identification of BfmR, a response regulator involved in biofilm development, as a target for a 2-aminoimidazole-based antibiofilm agent. Biochemistry 51:9776–9778. doi:10.1021/bi3015289. 23186243 PMC3567222

[14] David SA, Bechtel B, Annaiah C, Mathan VI, Balaram P. 1994. Interaction of cationic amphiphilic drugs with lipid A: implications for development of endotoxin antagonists. Biochim Biophys Acta 1212:167–175. doi:10.1016/0005-2760(94)90250-X. 8180242

[15] Harding CM, Hennon SW, Feldman MF. 2018. Uncovering the mechanisms of *Acinetobacter baumannii* virulence. Nat Rev Microbiol 16:91–102. doi:10.1038/nrmicro.2017.148. 29249812 PMC6571207

[16] Geisinger E, Huo W, Hernandez-Bird J, Isberg RR. 2019. *Acinetobacter baumannii*: envelope determinants that control drug resistance, virulence, and surface variability. Annu Rev Microbiol 73:481–506. doi:10.1146/annurev-micro-020518-115714. 31206345

[17] Geisinger E, Mortman NJ, Dai Y, Cokol M, Syal S, Farinha A, Fisher DG, Tang AY, Lazinski DW, Wood S, Anthony J, van Opijnen T, Isberg RR. 2020. Antibiotic susceptibility signatures identify potential antimicrobial targets in the *Acinetobacter baumannii* cell envelope. Nat Commun 11:4522. doi:10.1038/s41467-020-18301-2. 32908144 PMC7481262

[18] Knight D, Dimitrova DD, Rudin SD, Bonomo RA, Rather PN. 2016. Mutations decreasing intrinsic beta-lactam resistance are linked to cell division in the nosocomial pathogen *Acinetobacter baumannii*. Antimicrob Agents Chemother 60:3751–3758. doi:10.1128/AAC.00361-16. 27067318 PMC4879375

[19] Jacobs AC, Thompson MG, Black CC, Kessler JL, Clark LP, McQueary CN, Gancz HY, Corey BW, Moon JK, Si Y, Owen MT, Hallock JD, Kwak YI, Summers A, Li CZ, Rasko DA, Penwell WF, Honnold CL, Wise MC, Waterman PE, Lesho EP, Stewart RL, Actis LA, Palys TJ, Craft DW, Zurawski DV. 2014. AB5075, a highly virulent isolate of *Acinetobacter baumannii*, as a model strain for the evaluation of pathogenesis and antimicrobial treatments. mBio 5:e01076-14. doi:10.1128/mBio.01076-14. 24865555 PMC4045072

[20] Gallagher LA, Ramage E, Weiss EJ, Radey M, Hayden HS, Held KG, Huse HK, Zurawski DV, Brittnacher MJ, Manoil C. 2015. Resources for genetic and genomic analysis of emerging pathogen *Acinetobacter baumannii*. J Bacteriol 197:2027–2035. doi:10.1128/JB.00131-15. 25845845 PMC4438207

[21] Héritier C, Poirel L, Fournier P-E, Claverie J-M, Raoult D, Nordmann P. 2005. Characterization of the naturally occurring oxacillinase of *Acinetobacter baumannii*. Antimicrob Agents Chemother 49:4174–4179. doi:10.1128/AAC.49.10.4174-4179.2005. 16189095 PMC1251506

[22] Rodríguez-Martínez J-M, Poirel L, Nordmann P, 2010. Genetic and functional variability of AmpC-type beta-lactamases from *Acinetobacter baumannii*. Antimicrob Agents Chemother 54:4930–4933. doi:10.1128/AAC.00427-10. 20713667 PMC2976133

[23] Bonnin RA, Nordmann P, Potron A, Lecuyer H, Zahar JR, Poirel L. 2011. Carbapenem-hydrolyzing GES-type extended-spectrum beta-lactamase in *Acinetobacter baumannii*. Antimicrob Agents Chemother 55:349–354. doi:10.1128/AAC.00773-10. 20956589 PMC3019676

[24] Poirel L, Nordmann P. 2006. Carbapenem resistance in *Acinetobacter baumannii*: mechanisms and epidemiology. Clin Microbiol Infect 12:826–836. doi:10.1111/j.1469-0691.2006.01456.x. 16882287

[25] Turton JF, Ward ME, Woodford N, Kaufmann ME, Pike R, Livermore DM, Pitt TL. 2006. The role of ISAba1 in expression of OXA carbapenemase genes in *Acinetobacter baumannii*. FEMS Microbiol Lett 258:72–77. doi:10.1111/j.1574-6968.2006.00195.x. 16630258

[26] Powers MJ, Trent MS. 2018. Expanding the paradigm for the outer membrane: *Acinetobacter baumannii* in the absence of endotoxin. Mol Microbiol 107:47–56. doi:10.1111/mmi.13872. 29114953 PMC5740007

[27] Boll JM, Crofts AA, Peters K, Cattoir V, Vollmer W, Davies BW, Trent MS. 2016. A penicillin-binding protein inhibits selection of colistin-resistant, lipooligosaccharide-deficient *Acinetobacter baumannii*. Proc Natl Acad Sci USA 113:E6228–E6237. doi:10.1073/pnas.1611594113. 27681618 PMC5068286

[28] Nikaido H. 2009. Multidrug resistance in bacteria. Annu Rev Biochem 78:119–146. doi:10.1146/annurev.biochem.78.082907.145923. 19231985 PMC2839888

[29] Tipton KA, Chin CY, Farokhyfar M, Weiss DS, Rather PN. 2018. Role of capsule in resistance to disinfectants, host antimicrobials, and desiccation in *Acinetobacter baumannii*. Antimicrob Agents Chemother 62:e01188-18. doi:10.1128/AAC.01188-18. 30297362 PMC6256795

[30] Chin CY, Tipton KA, Farokhyfar M, Burd EM, Weiss DS, Rather PN. 2018. A high-frequency phenotypic switch links bacterial virulence and environmental survival in *Acinetobacter baumannii*. Nat Microbiol 3:563–569. doi:10.1038/s41564-018-0151-5. 29693659 PMC5921939

[31] Anderson SE, Sherman EX, Weiss DS, Rather PN. 2018. Aminoglycoside heteroresistance in *Acinetobacter baumannii* AB5075. mSphere 3:e00271-18. doi:10.1128/mSphere.00271-18. 30111627 PMC6094062

[32] Tipton KA, Dimitrova D, Rather PN. 2015. Phase-variable control of multiple phenotypes in *Acinetobacter baumannii* strain AB5075. J Bacteriol 197:2593–2599. doi:10.1128/JB.00188-15. 26013481 PMC4518826

[33] Wu X, Chavez JD, Schweppe DK, Zheng C, Weisbrod CR, Eng JK, Murali A, Lee SA, Ramage E, Gallagher LA, Kulasekara HD, Edrozo ME, Kamischke CN, Brittnacher MJ, Miller SI, Singh PK, Manoil C, Bruce JE. 2016. *In vivo* protein interaction network analysis reveals porin-localized antibiotic inactivation in *Acinetobacter baumannii* strain AB5075. Nat Commun 7:13414. doi:10.1038/ncomms13414. 27834373 PMC5114622

[34] Gallagher LA, Lee SA, Manoil C. 2017. Importance of core genome functions for an extreme antibiotic resistance trait. mBio 8:e01655-17. doi:10.1128/mBio.01655-17. 29233894 PMC5727411

[35] Perez-Varela M, Tierney ARP, Kim JS, Vazquez-Torres A, Rather P. 2020. Characterization of RelA in *Acinetobacter baumannii*. J Bacteriol 202:e00045-20. doi:10.1128/JB.00045-20. 32229531 PMC7253615

[36] Williams CL, Neu HM, Alamneh YA, Reddinger RM, Jacobs AC, Singh S, Abu-Taleb R, Michel SLJ, Zurawski DV, Merrell DS. 2020. Characterization of *Acinetobacter baumannii* copper resistance reveals a role in virulence. Front Microbiol 11:16. doi:10.3389/fmicb.2020.00016. 32117089 PMC7015863

[37] Elliott KT, Neidle EL. 2011. *Acinetobacter baylyi* ADP1: transforming the choice of model organism. IUBMB Life 63:1075–1080. doi:10.1002/iub.530. 22034222

[38] Machovina MM, Mallinson SJB, Knott BC, Meyers AW, Garcia-Borras M, Bu LT, Gado JE, Oliver A, Schmidt GP, Hinchen DJ, Crowley MF, Johnson CW, Neidle EL, Payne CM, Houk KN, Beckham GT, McGeehan JE, DuBois JL. 2019. Enabling microbial syringol conversion through structure-guided protein engineering. Proc Natl Acad Sci USA 116:13970–13976. doi:10.1073/pnas.1820001116. 31235604 PMC6628648

[39] Suarez GA, Dugan KR, Renda BA, Leonard SP, Gangavarapu LS, Barrick JE. 2020. Rapid and assured genetic engineering methods applied to *Acinetobacter baylyi* ADP1 genome streamlining. Nucleic Acids Res 48:4585–4600. doi:10.1093/nar/gkaa204. 32232367 PMC7192602

[40] Hsueh SC, Lee YJ, Huang YT, Liao CH, Tsuji M, Hsueh PR. 2019. *In vitro* activities of cefiderocol, ceftolozane/tazobactam, ceftazidime/avibactam, and other comparative drugs against imipenem-resistant *Pseudomonas aeruginosa* and *Acinetobacter baumannii*, and *Stenotrophomonas maltophilia*, all associated with bloodstream infections in Taiwan. J Antimicrob Chemother 74:380–386. doi:10.1093/jac/dky425. 30357343

[41] El Hafi B, Rasheed SS, Abou Fayad AG, Araj GF, Matar GM. 2019. Evaluating the efficacies of carbapenem/beta-lactamase inhibitors against carbapenem-resistant Gram-negative bacteria *in vitro* and *in vivo*. Front Microbiol 10:933. doi:10.3389/fmicb.2019.00933. 31114565 PMC6503214

[42] Brackett CM, Melander RJ, An IH, Krishnamurthy A, Thompson RJ, Cavanagh J, Melander C. 2014. Small-molecule suppression of beta-lactam resistance in multidrug-resistant gram-negative pathogens. J Med Chem 57:7450–7458. doi:10.1021/jm501050e. 25137478

[43] Shippy DC, Fadl AA. 2015. RNA modification enzymes encoded by the gid operon: implications in biology and virulence of bacteria. Microb Pathog 89:100–107. doi:10.1016/j.micpath.2015.09.008. 26427881

[44] Frech C, Wunderlich M, Glockshuber R, Schmid FX. 1996. Competition between DsbA-mediated oxidation and conformational folding of RTEM1 beta-lactamase. Biochemistry 35:11386–11395. doi:10.1021/bi9608525. 8784194

[45] Lo Sciuto A, Martorana AM, Fernandez-Pinar R, Mancone C, Polissi A, Imperi F. 2018. *Pseudomonas aeruginosa* LptE is crucial for LptD assembly, cell envelope integrity, antibiotic resistance and virulence. Virulence 9:1718–1733. doi:10.1080/21505594.2018.1537730. 30354941 PMC7204523

[46] Bos MP, Tommassen J. 2011. The LptD chaperone LptE is not directly involved in lipopolysaccharide transport in *Neisseria meningitidis*. J Biol Chem 286:28688–28696. doi:10.1074/jbc.M111.239673. 21705335 PMC3190676

[47] Crepin S, Ottosen EN, Chandler CE, Sintsova A, Ernst RK, Mobley HLT. 2020. The UDP-GalNAcA biosynthesis genes *gna-gne2* are required to maintain cell envelope integrity and *in vivo* fitness in multidrug resistant *Acinetobacter baumannii*. Mol Microbiol 113:153–860. doi:10.1111/mmi.14407. 31680352 PMC7007346

[48] Jacobs AC, Blanchard CE, Catherman SC, Dunman PM, Murata Y. 2014. A ribonuclease T2 family protein modulates *Acinetobacter baumannii* abiotic surface colonization. PLoS One 9:e85729. doi:10.1371/journal.pone.0085729. 24489668 PMC3904860

[49] Mu X, Wang N, Li X, Shi K, Zhou Z, Yu Y, Hua X. 2016. The effect of colistin resistance-associated mutations on the fitness of *Acinetobacter baumannii*. Front Microbiol 7:1715. doi:10.3389/fmicb.2016.01715. 27847502 PMC5088200

[50] Lonergan ZR, Nairn BL, Wang J, Hsu YP, Hesse LE, Beavers WN, Chazin WJ, Trinidad JC, VanNieuwenhze MS, Giedroc DP, Skaar EP. 2019. An *Acinetobacter baumannii*, zinc-regulated peptidase maintains cell wall integrity during immune-mediated nutrient sequestration. Cell Rep 26:2009–2018. doi:10.1016/j.celrep.2019.01.089. 30784584 PMC6441547

[51] Beceiro A, Pérez-Llarena FJ, Pérez A, Tomás MDM, Fernández A, Mallo S, Villanueva R, Bou G. 2007. Molecular characterization of the gene encoding a new AmpC beta-lactamase in *Acinetobacter baylyi*. J Antimicrob Chemother 59:996–1000. doi:10.1093/jac/dkm070. 17403709

[52] Simpson BW, Trent MS. 2019. Pushing the envelope: LPS modifications and their consequences. Nat Rev Microbiol 17:403–416. doi:10.1038/s41579-019-0201-x. 31142822 PMC6913091

[53] Boll JM, Tucker AT, Klein DR, Beltran AM, Brodbelt JS, Davies BW, Trent MS. 2015. Reinforcing lipid A acylation on the cell surface of *Acinetobacter baumannii* promotes cationic antimicrobial peptide resistance and desiccation survival. mBio 6:e00478-15. doi:10.1128/mBio.00478-15. 25991684 PMC4442142

[54] Moffatt JH, Harper M, Harrison P, Hale JD, Vinogradov E, Seemann T, Henry R, Crane B, St Michael F, Cox AD, Adler B, Nation RL, Li J, Boyce JD. 2010. Colistin resistance in *Acinetobacter baumannii* is mediated by complete loss of lipopolysaccharide production. Antimicrob Agents Chemother 54:4971–4977. doi:10.1128/AAC.00834-10. 20855724 PMC2981238

[55] Powers MJ, Trent MS. 2018. Phospholipid retention in the absence of asymmetry strengthens the outer membrane permeability barrier to last-resort antibiotics. Proc Natl Acad Sci USA 115:E8518–E8527. doi:10.1073/pnas.1806714115. 30087182 PMC6130378

[56] Miller BW, Lim AL, Lin Z, Bailey J, Aoyagi KL, Fisher MA, Barrows LR, Manoil C, Schmidt EW, Haygood MG. 2021. Shipworm symbiosis ecology-guided discovery of an antibiotic that kills colistin-resistant *Acinetobacter*. Cell Chem Biol 28:1628–1637. doi:10.1016/j.chembiol.2021.05.003. 34146491 PMC8605984

[57] Farha MA, Brown ED. 2016. Strategies for target identification of antimicrobial natural products. Nat Prod Rep 33:668–680. doi:10.1039/c5np00127g. 26806527

[58] Ferrières L, Hémery Glle, Nham T, Guérout A-M, Mazel D, Beloin C, Ghigo J-M. 2010. Silent mischief: bacteriophage Mu insertions contaminate products of *Escherichia coli* random mutagenesis performed using suicidal transposon delivery plasmids mobilized by broad-host-range RP4 conjugative machinery. J Bacteriol 192:6418–6427. doi:10.1128/JB.00621-10. 20935093 PMC3008518

[59] Bailey J, Cass J, Gasper J, Ngo ND, Wiggins P, Manoil C. 2019. Essential gene deletions producing gigantic bacteria. PLoS Genet 15:e1008195. doi:10.1371/journal.pgen.1008195. 31181062 PMC6586353

[60] Gallagher LA, Bailey J, Manoil C. 2020. Ranking essential bacterial processes by speed of mutant death. Proc Natl Acad Sci USA 117:18010–18017. doi:10.1073/pnas.2001507117. 32665440 PMC7395459

[61] Gallagher LA, Shendure J, Manoil C. 2011. Genome-scale identification of resistance functions in *Pseudomonas aeruginosa* using Tn-seq. mBio 2:e00315-10–e00310. 21253457 10.1128/mBio.00315-10 PMC3023915

