# Supplementary figures and images for: Genetic Dissection of Antibiotic Adjuvant Activity
Source: mBio. 2022 Jan 18;13(1):e03084-21. doi: 10.1128/mbio.03084-21 (PMC8764523; doi:10.1128/mbio.03084-21)

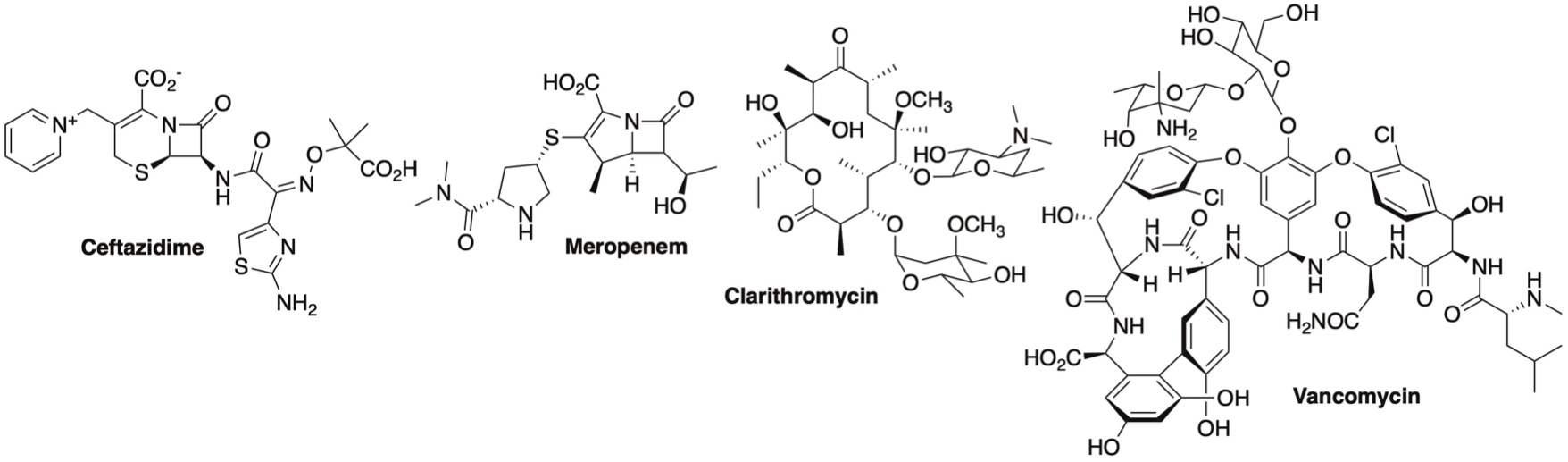

Supplement: FIG S1 [file mbio.03084-21-sf001.tif]
