# Supplementary material for: Genetic Dissection of Antibiotic Adjuvant Activity
Source: mBio. 2022 Jan 18;13(1):e03084-21. doi: 10.1128/mbio.03084-21 (PMC8764523; doi:10.1128/mbio.03084-21)
Supplement: TABLE S1 [file mbio.03084-21-st001.docx]

| **Table S1. Ceftazidime sensitive mutants identified by Tn-seq.** The genes with the strongest mutant ceftazidime sensitivity phenotypes based on loss after growth in normally sub-inhibitory ceftazidime are listed at the top. Genes with weaker mutant meropenem sensitivity phenotypes or encoding β-lactamases are included below. The top ceftazidime sensitivity genes correspond to those significantly depleted (P<0.05) in the assays at 32 µg/ml and 64 µg/ml ceftazidime, with a minimum of 100 reads and length of at least 100 residues. A gene that has scored as essential in most other assays and narrowly exceeded the minimum read cut-off here (*nrdB*) was not included. | | | | | | |
| --- | --- | --- | --- | --- | --- | --- |
| **Locus** | **Gene** | **Product** | **Read recovery**  **(log +ceftazidime /log –ceftazidime)** | | | |
|  |  |  | **32 µg/ml** | | **64 µg/ml** | **128 µg/ml** |
| **Top ceftazidime sensitive mutants** | | | | | | |
| ABUW_4052 | *bla_GES-14_* | β-lactamase GES-14 | | <0.6 | <0.6 | <0.6 |
| ABUW_4001 | *repA* | Plasmid p1 replicase | | <0.6 | <0.6 | <0.6 |
| ABUW_1537 | *gidA* | tRNA modification | | <0.6 | <0.6 | 0.97 |
| **Weaker ceftazidime sensitive mutants and other ß-lactamase mutants** | | | | | | |
| ABUW_3360 | *lptE* | Lipopolysaccharide transport | | 1.09 | 0.90 | <0.6 |
| ABUW_3846 | *dsbA* | Thiol:disulfide interchange | | 0.88 | 0.90 | 0.64 |
|  | | | | | | |
| ABUW_0563 | *bla_OXA-23_* | β-lactamase OXA-23 | | 0.97 | 0.97 | 0.97 |
| ABUW_1194 | *ampC* | β-lactamase ADC7 | | 1.02 | 1.01 | 0.92 |
| ABUW_2300 | *bla_OXA-69_* | β-lactamase OXA-69 | | 0.99 | 1.03 | 1.03 |
