# Supplementary material for: Genetic Dissection of Antibiotic Adjuvant Activity
Source: mBio. 2022 Jan 18;13(1):e03084-21. doi: 10.1128/mbio.03084-21 (PMC8764523; doi:10.1128/mbio.03084-21)
Supplement: TABLE S2 [file mbio.03084-21-st002.docx]

| **Table S2. Meropenem sensitive mutants identified by Tn-seq.** The genes and corresponding sequence read recoveries for growth on LB agar containing meropenem relative to agar without meropenem are shown for seven independent Tn-seq assays at the meropenem concentrations indicated. Mutants were grouped into four classes based on meropenem sensitivity with the following probability cutoffs: class 1, depleted in 2/2 4 µg/ml assays; class 2, depleted in 1/2 4 µg/ml and ≥2/3 6 µg/ml assays; class 3, depleted in ≥2/3 6 µg/ml assays only; class 4, depleted in 1-2/3 6 µg/ml assays only but associated with peptidoglycan synthesis or protein secretion. The probability cutoffs used were P<0.01 (4 µg/ml meropenem Trial 1, and 6 µg/ml meropenem Trials 1 and 2), and P<0.05 (4 µg/ml meropenem Trial 2 and 6 µg/ml meropenem Trial 3). Several genes exhibited meropenem sensitive mutant phenotypes but were not included because they were slow growing in the absence of antibiotic and missed the essentiality cutoff (*ompA*, *ompR*, *rpoH* and *rlpA*). | | | | | | | | | | | | |
| --- | --- | --- | --- | --- | --- | --- | --- | --- | --- | --- | --- | --- |
| **Locus** | **Gene** | **Product** | **Class** | | **Read recovery (log +meropenem /log –meropenem)** | | | | | | | |
|  | | | | | **1 µg/ml** | **2 µg/ml** | **4 µg/ml** | | **6 µg/ml** | | | |
|  |  |  |  |  |  | | **Trial 1** | **Trial 2** | | **Trial 1** | **Trial 2** | **Trial 3** |
| ABUW_3360 | *lptE* | Lipooligosaccharide transport | | 1 | <0.14 | <0.14 | <0.12 | <0.14 | | <0.12 | <0.11 | <0.14 |
| ABUW_0563 | *bla_OXA-23_* | b-lactamase OXA-23 | | 1 | 1.0 | 0.90 | 0.28 | <0.10 | | 0.29 | 0.22 | 0.09 |
| ABUW_3638 | *pbpG* | D-alanyl-D-alanine carboxypeptidase | | 1 | 0.96 | 0.99 | 0.84 | 0.32 | | 0.60 | 0.17 | 0.36 |
| ABUW_3740 | *znuA* | High affinity Zn transport protein | | 1 | 1.02 | 1.01 | 0.71 | 0.56 | | 0.14 | 0.09 | <0.12 |
| ABUW_3830 | *gna* | UDP-*N*-acetylglucosamine C-6 dehydrogenase | | 1 | 0.98 | 0.78 | 0.54 | 0.58 | | 0.60 | 0.53 | <0.14 |
| ABUW_3846 | *dsbA* | Thiol:disulfide interchange protein | | 1 | 0.77 | 0.88 | 0.73 | 0.31 | | 0.78 | 0.59 | 0.53 |
| ABUW_0115 | *dsbB* | Disulfide bond formation protein | | 2 | 1.01 | 0.95 | 0.86 | 0.76 | | 0.88 | 0.77 | 0.82 |
| ABUW_0460 | *-* | Hypothetical protein | | 2 | 0.84 | 0.87 | 0.99 | 0.53 | | 0.81 | 0.78 | 0.45 |
| ABUW_0466 | *-* | Hypothetical protein | | 2 | 0.97 | 0.94 | 0.91 | 0.85 | | 0.53 | 0.41 | 0.58 |
| ABUW_0988 | *rpoE* | RNA polymerase sigma factor | | 2 | 0.97 | 0.93 | 0.90 | 0.93 | | 0.82 | 0.79 | 0.76 |
| ABUW_1134 | *ampG* | Muropeptide transporter | | 2 | 0.96 | 0.92 | 0.75 | 0.74 | | 0.34 | 0.28 | 0.41 |
| ABUW_1740 | *rseP* | Inner membrane zinc metalloprotease; rpoE activator | | 2 | 0.97 | 0.90 | 0.87 | 0.92 | | 0.69 | 0.63 | 0.49 |
| ABUW_3447 | *lpxL* | Lipid A biosynthesis acyltransferase | | 2 | 1.00 | 1.00 | 0.86 | 0.95 | | 0.84 | 0.58 | 0.78 |
| ABUW_3448 | *lpsB* | Lipooligosaccharide core biosynthesis glycosyl transferase | | 2 | 0.95 | 0.65 | 0.57 | 0.80 | | 0.49 | 0.44 | 0.54 |
| ABUW_3741 | *zur* | Transcriptional regulator | | 2 | 1.02 | 1.02 | 0.73 | 0.64 | | 0.46 | 0.49 | 0.27 |
| ABUW_3742 | *znuC* | High affinity Zn transport protein | | 2 | 1.03 | 1.05 | 0.81 | 0.64 | | 0.39 | 0.38 | 0.36 |
| ABUW_3743 | *znuB* | High affinity Zn transport protein | | 2 | 1.02 | 1.02 | 0.81 | 0.69 | | 0.34 | 0.24 | 0.11 |
| ABUW_3825 | *wzy* | Capsule polymerase | | 2 | 1.14 | 1.09 | 0.86 | 1.08 | | 0.79 | 0.83 | 1.09 |
| ABUW_3832 | *wzb* | Polysaccharide export | | 2 | 0.94 | 0.95 | 0.90 | 0.97 | | 0.87 | 0.85 | 0.92 |
| ABUW_3833 | *wzc* | Polysaccharide export | | 2 | 1.05 | 1.03 | 0.90 | 1.00 | | 0.82 | 0.81 | 0.92 |
| ABUW_2268 | *surA* | Peptidyl-prolyl cis-trans isomerase | | 3 | 0.99 | 1.00 | 0.97 | 0.99 | | 0.78 | 0.57 | 0.56 |
| ABUW_3041 | *algC* | Phosphomannomutase | | 3 | 0.99 | 1.00 | 0.98 | 0.97 | | 0.84 | 0.58 | 0.78 |
| ABUW_3180 | *bfmS* | Two-component system sensor kinase | | 3 | 0.93 | 0.98 | 0.96 | 1.01 | | 0.62 | 0.54 | 0.81 |
| ABUW_3627 | *dksA* | DnaK suppressor | | 3 | 1.08 | 0.97 | 0.98 | 0.88 | | 0.88 | 0.65 | 0.66 |
| ABUW_3632 | *feoB* | Ferrous iron transport | | 3 | 1.04 | 1.01 | 0.97 | 0.99 | | 0.64 | 0.43 | 0.59 |
| ABUW_3820 | *gdr* | UDP-Glc*p*NAc 4,6-dehydratase | | 3 | 1.01 | 1.03 | 0.92 | 0.98 | | 0.73 | 0.79 | 0.80 |
| ABUW_3821 | *qhbB* | UDP-D-QuipNAc4 synthesis pathway | | 3 | 1.04 | 1.02 | 0.92 | 1.00 | | 0.62 | 0.29 | 0.67 |
| ABUW_3822 | *qhbA* | UDP-D-QuipNAc4 synthesis pathway | | 3 | 0.97 | 1.02 | 0.95 | 0.95 | | 0.83 | 0.69 | 0.74 |
| ABUW_3823 | *itrA1* | Initiating transferase | | 3 | 1.01 | 1.00 | 0.95 | 1.00 | | 0.82 | 0.58 | 0.80 |
| ABUW_3824 | *gtr52* | Glycosyltransferase | | 3 | 0.99 | 0.99 | 0.98 | 0.98 | | 0.84 | 0.71 | 0.88 |
| ABUW_3828 | *mnaB* | UDP-N-acetyl-D-mannosamine dehydrogenase | | 3 | 0.96 | 1.00 | 0.94 | 1.00 | | 0.77 | 0.68 | 0.78 |
| ABUW_3837 | *ampD* | N-acetylmuramoyl-L-alanine amidase | | 3 | 0.98 | 1.00 | 0.93 | 0.96 | | 0.80 | 0.74 | 0.87 |
| ABUW_0154 | *mepM* | Murein peptidase | | 4 | 1.00 | 0.98 | 1.01 | 1.01 | | 0.92 | 0.74 | 0.86 |
| ABUW_0532 | *yajC* | Preprotein translocase | | 4 | 0.77 | 0.84 | 0.93 | 0.79 | | 0.91 | 0.55 | 0.85 |
| ABUW_3408 | *gcf* | Giant cell formation | | 4 | 1.03 | 0.99 | 1.00 | 0.96 | | 0.98 | 0.80 | 0.89 |
| ABUW_4052 | *bla_GES-14_* | b-lactamase GES-14 | | 4 | 0.84 | 0.96 | 1.01 | 0.90 | | 0.94 | 0.70 | 0.82 |
| ABUW_1194 | *ampC* | b-lactamase ADC7 | | – | 1.00 | 1.00 | 0.99 | 0.99 | | 0.99 | 1.00 | 0.99 |
| ABUW_2300 | *bla_OXA-69_* | b -lactamase OXA-69 | | – | 1.01 | 1.04 | 1.00 | 1.04 | | 1.00 | 0.99 | 1.05 |
