# Supplementary material for: Genetic Dissection of Antibiotic Adjuvant Activity
Source: mBio. 2022 Jan 18;13(1):e03084-21. doi: 10.1128/mbio.03084-21 (PMC8764523; doi:10.1128/mbio.03084-21)
Supplement: TABLE S3 [file mbio.03084-21-st003.docx]

| **Table S3. Vancomycin and clarithromycin sensitive mutants identified using Tn-seq.** Genes with greatest depletion of transposon insertion reads following growth in the presence of normally sub-inhibitory vancomycin or clarithromycin are shown. Genes identified in both screens are in boldface. The genes listed correspond to those showing strongest mutant depletion (lowest log sequence read ratios) after growth in the levels of antibiotic shown relative to no antibiotic (≥2/3 P<0.01). vanc, vancomycin; clar, clarithromycin | | | | | | | |  |  |
| --- | --- | --- | --- | --- | --- | --- | --- | --- | --- |
|  |  |  | | | |  |  | |  |
| **Vancomycin** |  |  |  | | |  |  | |  |
| **Locus** | **Gene** | **Product** | **Reads (log + vanc/log – vanc)** | | |  |  | |  |
|  |  |  | **12 µg/ml** | **18 µg/ml** | **30 µg/ml** |  |  | |  |
| ABUW_0037 | dnaJ | Chaperone | 0.74 | <0.4 | 0.69 |  |  | |  |
| **ABUW_1207** | **glnA** | **Glutamine synthetase** | **<0.5** | **0.93** | **0.52** |  |  | |  |
| **ABUW_1242** | **rlpA** | **Rare lipoprotein A** | **0.62** | **0.73** | **<0.5** |  |  | |  |
| ABUW_1856 | - | Transcriptional regulator, MarR-family | <0.5 | 0.96 | <0.5 |  |  | |  |
| ABUW_2240 | - | Hypothetical protein | 0.60 | 0.95 | 0.56 |  |  | |  |
| ABUW_2610 | - | Hypothetical protein | 0.65 | <0.5 | 0.77 |  |  | |  |
| **ABUW_3360** | **lptE** | **LOS assembly** | **<0.5** | **<0.5** | **<0.5** |  |  | |  |
| ABUW_3393 | eda | Entner-Doudoroff aldolase | 0.69 | 1.0 | 0.45 |  |  | |  |
| ABUW_3447 | lpxL | Lipid A acyltransferase | 0.86 | 0.67 | 0.69 |  |  | |  |
| **ABUW_3448** | **lpsB** | **LOS core synthesis** | **0.42** | **0.51** | **<0.3** |  |  | |  |
| ABUW_3639 | gacA | Response regulator | <0.4 | 0.89 | <0.4 |  |  | |  |
| ABUW_3652 | ribD | Riboflavin biosynthesis | 0.94 | <0.50 | 0.58 |  |  | |  |
| **ABUW_3826** | **gtr51** | **Glycosyl transferase** | **<0.4** | **0.68** | **0.41** |  |  | |  |
| **ABUW_3830** | **gna** | **UDP-N-acetylglucosamine C-6 dehydrogenase** | **0.45** | **0.42** | **0.32** |  |  | |  |
| ABUW_3831 | wza | Polysaccharide export | 0.81 | 0.73 | 0.72 |  |  | |  |
|  |  |  |  |  |  |  |  | |  |
|  |  |  |  |  |  |  |  | |  |
| **Clarithromycin** | |  |  |  |  |  |  | |  |
| **Locus** | **Gene** | **Product** | **Reads (log + clar /log – clar)** | | |  |  | |  |
|  |  |  | **0.4 µg/ml** | **0.6 µg/ml** | **1.0 µg/ml** |  |  | |  |
| **ABUW_1207** | **glnA** | **Glutamine synthetase** | **<0.5** | **<0.5** | **<0.5** |  |  | |  |
| **ABUW_1242** | **rlpA** | **Rare lipoprotein A** | **0.76** | **0.49** | **0.56** |  |  | |  |
| ABUW_1516 | - | Hypothetical protein | 0.64 | 0.91 | 0.75 |  |  | |  |
| ABUW_2905 | estR | Transcriptional regulator | <0.5 | <0.5 | 1.1 |  |  | |  |
| **ABUW_3360** | **lptE** | **LOS assembly** | **0.72** | **<0.4** | **<0.4** |  |  | |  |
| **ABUW_3448** | **lpsB** | **LOS core synthesis** | **0.42** | **0.53** | **<0.3** |  |  | |  |
| **ABUW_3826** | **gtr51** | **Glycosyl transferase** | **<0.5** | **<0.5** | **<0.5** |  |  | |  |
| **ABUW_3830** | **gna** | **UDP-N-acetylglucosamine C-6 dehydrogenase** | **0.55** | **0.29** | **0.45** |  |  | |  |
| ABUW_3846 | dsbA | Thiol:disulfide interchange protein | <0.5 | 0.81 | 0.57 |  |  | |  |
