# Supplementary material for: Genetic Dissection of Antibiotic Adjuvant Activity
Source: mBio. 2022 Jan 18;13(1):e03084-21. doi: 10.1128/mbio.03084-21 (PMC8764523; doi:10.1128/mbio.03084-21)
Supplement: TABLE S4 [file mbio.03084-21-st004.docx]

| **Table S4. Mutants sensitive to adjuvant AI-1 in Tn-seq.** Genes showing depletion of transposon insertion sequence reads following growth in the presence of normally sub-inhibitory adjuvant AI-1 (LB containing 20 µM AI-1) in two independent trials are listed. The genes listed correspond to those showing greatest mutant depletion after growth with adjuvant relative to without adjuvant ( P<0.01 in both trials) for the log sequence read ratios in Tn-seq. Genes ABUW_3816 to ABUW_3832 are K locus genes involved in extracellular capsule production. | | | | |
| --- | --- | --- | --- | --- |
| **Locus** | **Gene** | **Function** | **Reads (log +AI-1/ log –AI-1)** | |
|  |  |  | **Trial 1** | **Trial 2** |
| ABUW_0135 | – | Hypothetical | 0.39 | 0.54 |
| ABUW_0383 | mlaF | Phospholipid transport | <0.3 | 0.59 |
| ABUW_0385 | mlaD | “ | <0.3 | 0.67 |
| ABUW_0386 | mlaC | “ | <0.3 | 0.43 |
| ABUW_0591 | lpsC | LOS outer core glycosyltransferase | <0.4 | <0.4 |
| ABUW_0592 | – | “ | <0.3 | <0.3 |
| ABUW_0594 | – | “ | 0.55 | 0.35 |
| ABUW_1207 | glnA | Glutamine synthetase | <0.5 | <0.5 |
| ABUW_1242 | rlpA | Rare lipoprotein A | <0.5 | <0.5 |
| ABUW_1324 | – | Hypothetical | <0.5 | 0.46 |
| ABUW_1375 | rpoH | Alternative sigma factor | 0.37 | 0.60 |
| ABUW_1608 | miaA | tRNA delta(2)-isopentenylpyrophosphate transferase | <0.4 | 0.48 |
| ABUW_2672 | – | Hypothetical | <0.5 | 0.58 |
| ABUW_3259 | mlaA | Phospholipid transport | <0.3 | <0.3 |
| ABUW_3302 | relA | ppGpp synthetase I | <0.3 | 0.66 |
| ABUW_3369 | – | Rodanese domain | 0.34 | <0.3 |
| ABUW_3448 | lpsB | LOS outer core glycosyltransferase | <0.3 | 0.41 |
| ABUW_3816 | gne1 | UDP-N-acetylglucosaminuronic acid C-4 epimerase | <0.3 | <0.3 |
| ABUW_3820 | gdr | UDP-GlcpNAc 4,6-dehydratase | <0.3 | 0.35 |
| ABUW_3821 | qhbB | UDP-D-QuipNAc4NR | <0.3 | 0.43 |
| ABUW_3822 | qhbA | UDP-D-QuipNAc4NR | <0.4 | <0.4 |
| ABUW_3823 | itrA1 | initiating transferase | 0.36 | 0.26 |
| ABUW_3824 | gtr52 | Glycosyltransferase | 0.23 | 0.38 |
| ABUW_3825 | wzy | Polymerase | <0.4 | <0.3 |
| ABUW_3830 | gna | UDP-N-acetylglucosamine C-6 dehydrogenase | 0.23 | <0.3 |
| ABUW_3831 | wza | Polysaccharide export | <0.4 | <0.3 |
| ABUW_3832 | wzb | “ | <0.4 | <0.5 |
