# Supplementary material for: Genetic Dissection of Antibiotic Adjuvant Activity
Source: mBio. 2022 Jan 18;13(1):e03084-21. doi: 10.1128/mbio.03084-21 (PMC8764523; doi:10.1128/mbio.03084-21)
Supplement: TABLE S5 [file mbio.03084-21-st005.docx]

|  | | |  |  |  |  |  |
| --- | --- | --- | --- | --- | --- | --- | --- |
| **Table S5.** **Individual transposon insertion mutants from *A. baumannii* AB5075 transposon mutant library (20).** All alleles are chromosomal except blaGES-14::T26, which is situated on plasmid p1. | | | | | |  |  |
| **Strain** | **Allele** | **Locus** | **Genome location** | **Insertion location (bp)** |  |  |  |
| MAB00082 | dnaJ::T26 | ABUW_0037 | 45718 | 449(1113) |  |  |  |
| MAB00083 | dnaJ::T26 | ABUW_0037 | 45897 | 628(1113) |  |  |  |
| MAB00325 | dsbB::T26 | ABUW_0115 | 127929 | 56(f516) |  |  |  |
| MAB00326 | dsbB::T26 | ABUW_0115 | 127979 | 106(516) |  |  |  |
| MAB00713 | envZ::T26 | ABUW_0256 | 269752 | 888(1458) |  |  |  |
| MAB00717 | ompR::T26 | ABUW_0257 | 270920 | 502(765) |  |  |  |
| MAB00718 | ompR::T26 | ABUW_0257 | 271011 | 411(765) |  |  |  |
| MAB01224 | ABUW_0460::T26 | ABUW_0460 | 472639 | 51(672) |  |  |  |
| MAB01242 | ABUW_0466::T26 | ABUW_0466 | 479716 | 470(663) |  |  |  |
| MAB01241 | ABUW_0466::T26 | ABUW_0466 | 479566 | 320(663) |  |  |  |
| MAB01498 | oxa-23::T26 | ABUW_0563 | 563102 | 105(822) |  |  |  |
| MAB01499 | oxa-23::T26 | ABUW_0563 | 563118 | 121(822) |  |  |  |
| MAB01500 | oxa-23::T26 | ABUW_0563 | 563227 | 230(822) |  |  |  |
| MAB01670 | trpB1::T26 | ABUW_0617 | 618862 | 381(1230) |  |  |  |
| MAB01671 | trpB1::T26 | ABUW_0617 | 619151 | 670(1230) |  |  |  |
| MAB01772 | ompA::T26 | ABUW_0649 | 654325 | 103(1062) |  |  |  |
| MAB01775 | ompA::T26 | ABUW_0649 | 655165 | 943(1062) |  |  |  |
| MAB01891 | elsL::T26 | ABUW_0690 | 697961 | 239(504) |  |  |  |
| MAB01893 | elsL::T26 | ABUW_0690 | 698130 | 408(504) |  |  |  |
| MAB02671 | rpoE::T26 | ABUW_0988 | 1001421 | 409(615) |  |  |  |
| MAB02672 | rpoE::T26 | ABUW_0988 | 1001594 | 236(615) |  |  |  |
| MAB03057 | dacC::T26 | ABUW_1127 | 1149563 | 699(1149) |  |  |  |
| MAB03058 | dacC::T26 | ABUW_1127 | 1149721 | 541(1149) |  |  |  |
| MAB03078 | ampG::T26 | ABUW_1134 | 1157477 | 460(2190) |  |  |  |
| MAB03079 | ampG::T26 | ABUW_1134 | 1157796 | 779(2190) |  |  |  |
| MAB03238 | ldtG::T26 | ABUW_1189 | 1221086 | 864(1248) |  |  |  |
| MAB03239 | ldtG::T26 | ABUW_1189 | 1221330 | 620(1248) |  |  |  |
| MAB03240 | ldtG::T26 | ABUW_1189 | 1221582 | 368(1248) |  |  |  |
| MAB03359 | rlpA::T26 | ABUW_1242 | 1283813 | 552(618) |  |  |  |
| MAB03361 | rlpA::T26 | ABUW_1242 | 1284060 | 305(618) |  |  |  |
| MAB04094 | gidA::T26 | ABUW_1537 | 1523281 | 492(1881) |  |  |  |
| MAB04095 | gidA::T26 | ABUW_1537 | 1523615 | 826(1881) |  |  |  |
| MAB04096 | gidA::T26 | ABUW_1537 | 1524019 | 1230(1881) |  |  |  |
| MAB04573 | rseP::T26 | ABUW_1740 | 1737639 | 411(1356) |  |  |  |
| MAB04574 | rseP::T26 | ABUW_1740 | 1737903 | 675(1356) |  |  |  |
| MAB07523 | mrdA::T26 | ABUW_2876 | 2885443 | 443(2019) |  |  |  |
| MAB07524 | mrdA::T26 | ABUW_2876 | 2885708 | 708(2019) |  |  |  |
| MAB08787 | lptE::T26 | ABUW_3360 | 3399672 | 18(510) |  |  |  |
| MAB08788 | lptE::T26 | ABUW_3360 | 3399883 | 229(510) |  |  |  |
| MAB08789 | lptE::T26 | ABUW_3360 | 3400038 | 384(510) |  |  |  |
| MAB08791 | lptE::T26 | ABUW_3360 | 3400058 | 404(510) |  |  |  |
| MAB09045 | lpxL::T26 | ABUW_3447 | 3492031 | 314(936) |  |  |  |
| MAB09046 | lpxL::T26 | ABUW_3447 | 3492131 | 414(936) |  |  |  |
| MAB09049 | lpsB::T26 | ABUW_3448 | 3493251 | 333(1101) |  |  |  |
| MAB09052 | lpsB::T26 | ABUW_3448 | 3493660 | 742(1101) |  |  |  |
| MAB09537 | dksA::T26 | ABUW_3627 | 3686000 | 1(537) |  |  |  |
| MAB09565 | pbpG::T26 | ABUW_3638 | 3698242 | 277(1008) |  |  |  |
| MAB09566 | pbpG::T26 | ABUW_3638 | 3698379 | 140(1008) |  |  |  |
| MAB09850 | znuA::T26 | ABUW_3740 | 3796878 | 211(840) |  |  |  |
| MAB09849 | znuA::T26 | ABUW_3740 | 3796698 | 391(840) |  |  |  |
| MAB09855 | znuC::T26 | ABUW_3742 | 3798052 | 358(780) |  |  |  |
| MAB09854 | znuC::T26 | ABUW_3742 | 3797890 | 196(780) |  |  |  |
| MAB10089 | qhbB::T26 | ABUW_3821 | 3887308 | 868(1176) |  |  |  |
| MAB10090 | qhbB::T26 | ABUW_3821 | 3887719 | 457(1176) |  |  |  |
| MAB10122 | gna::T26 | ABUW_3830 | 3897717 | 319(1275) |  |  |  |
| MAB10123 | gna::T26 | ABUW_3830 | 3897879 | 157(1275) |  |  |  |
| MAB10171 | dsbA::T26 | ABUW_3846 | 3915141 | 336(618) |  |  |  |
| MAB10172 | dsbA::T26 | ABUW_3846 | 3915293 | 184(618) |  |  |  |
| MAB10174 | dsbA::T26 | ABUW_3846 | 3915413 | 64(618) |  |  |  |
| MAB10478 | blaGES-14::T26 | ABUW_4052 | 31309 | 378(864) |  |  |  |
