# Supplementary material for: Genetic Dissection of Antibiotic Adjuvant Activity
Source: mBio. 2022 Jan 18;13(1):e03084-21. doi: 10.1128/mbio.03084-21 (PMC8764523; doi:10.1128/mbio.03084-21)
Supplement: TABLE S6 [file mbio.03084-21-st006.docx]

| **Table S6. Oligonucleotide primers** | | |
| --- | --- | --- |
| **Name** | **Sequence** | **Application** |
| oxa23 up + | GGCGCTGCAGTGTTGCCATTCACCCGTTAAG | Construction MAB198, MAB200 |
| oxa23_5'_rev | ACACGACAAAAATAGATAACTCATTG |  |
| oxa23_3'_for | CAATGAGTTATCTATTTTTGTCGTGTAATATTATTTAAGAGCTTGACAGAAACTC |  |
| oxa23 down + | GGCGGAGCTCGATCTACCAAATGGACCCTGTAG |  |
| oxa23uconf | TTGGGCTTCTAGTGCCTTTAG |  |
| oxa23dconf | ACCAGCACTGACACCTCAG |  |
| blaGESup3 PstI | GGCGCTGCAGGCAGGGGTAGTGAATCCG | Construction MAB199, MAB200 |
| blaGES down BamHI | GGCGGGATCCGTAACTGCTCACCTTTTGCTG |  |
| blaGES 3' for | GATCACCATGCGCTTCATTCACAGCACGGACAAATAGTTGACG |  |
| blaGES rev | GTGAATGAAGCGCATGGTGATC |  |
| blaGESuconf | GCCAAAGCCTTAGCGTAGG |  |
| blaGESdconf | TTCCTTTCCTCGACACTACTG |  |
| bfmR up Pst | GGCGCTGCAGTCGATTTACAGTTGCTTGCAG | Construction MAB201 |
| bfmR down Sac | GGCGGAGCTCAAGTTTGCCATTTCGTCGC |  |
| bfmR 5 | TTCTTCTTGGCTCATATCATTGC |  |
| bfmR3 plus | GCAATGATATGAGCCAAGAAGAATTGTTTGTTAAAGAAACCAATGG |  |
| bfmRuconf | TCATGGTGGTAGCAATATCGC |  |
| bfmRdconf | GAGCCTCAATCAAACGCTG |  |
| lpxC seq5 | CGTACAATCTATCGAAAGGCAGTG | Sequencing LOS- strains including MAB202, MAB203 |
| lpxC seq3 | ACAAGAAATGTTACGTAGTGCCG |  |
| lpxA seq5 | TGAGTTGGTAATGCAGAAGCG |  |
| lpxA seq3 | GTAAGGCACCTCGAGCATTG |  |
| IS1up | GAATGCGAATGTCGGCTG | Construction MAY151, MAY153 |
| blaGES3 IS1 | CGCCCGTCTAACAATTCGATTGCATAAAAATGAAAGTAACTC |  |
| blaGES5 IS1 | CGTGCCTTCATCCGTTTCCATGTCCAGTCAATTGCTTTAATG |  |
| IS1down | CCTTACGCTGGTCTGACTTTG |  |
| blaGES5 | GAAACGGATGAAGGCACG | Amplification of ABUW_4052 |
| blaGES3 | CGAATTGTTAGACGGGCG |  |
| IS4up | AATACCACGCAACAACTGGG | Construction MAY152, MAY153 |
| IS4 5’ | ATGAACATCTTCAACATTTAGATCAAG |  |
| IS4 3’ | CATGCATTGCACGTTCG |  |
| IS4down | AATGCCAGATCTACACTGACG |  |
| oxa23 5 IS4 | CGAACGTGCAATGCATGAGCACTTTAAATGTGACTTGTTCC | Amplification of ABUW_0563 |
| oxa23 3 IS4 | CTTGATCTAAATGTTGAAGATGTTCATGATCACAACAACTAAAAGCACTG |  |
| mla up | CCGAGAGTTCTGCCATTTTGG | Construction MAY154 |
| mla 3 | CTTCATCGCTAGTAAGCTACC |  |
| mla 5 | GGTAGCTTACTAGCGATGAAGTGGAGTTGGATTATTCATAGC |  |
| mla down | ACCTCCTGCACGTTCTGG |  |
| pldA up | TTGGCTTGGAAGATGTACCTG |  |
| pldA 5 | CCCCTCAATAAATCTAAACGC |  |
| pldA 3 | GCGTTTAGATTTATTGAGGGGCTGATGAACTGGTATTGATCTG |  |
| pldA down | GAACCAGATCCTACTTGTCCTG |  |
| lptE up | TGGTGGTGTAGAACATGCG | Construction MAY156 |
| lptEaphAfor | ATGAGGTGCGACAGTTTCAAAAGGCTTGTTCCTCTTGACGCTTG |  |
| lptEaphArev | AAACTTGATGAATTGAATTGATTCCAAGCCTCATCTCTCAGCCTTAAGGG |  |
| lptE down | AGGCAAAGTAAGAAGGACTGG |  |
| lpxLup | TGTCAGTGCAATTCCTGAAGG | Construction MAY157 |
| lpxLaphAfor | ATGAGGTGCGACAGTTTCAAAAGGCTGAATTAGGGGGTGAC |  |
| lpxLaphArev | AAACTTGATGAATTGAATTGATTCCAAGCGATAAAGCTATAATGATTTTAAAG |  |
| lpxL down | CGAGTAATACGTCCAGGCAAG |  |
| lpxM up | GTCAGCCACACCCAGTTG | Construction MAY158 |
| lpxMaphAfor | ATGAGGTGCGACAGTTTCAAAAGAAACGATACATAAAGCTGCGTG |  |
| lpxMaphArev | AAACTTGATGAATTGAATTGATTCCAAGCCGTCTTAACGCTGCAACC |  |
| lpxM down | ACAGCAGTTTCATCAGACTCG |  |
